# Supplementary material for: Do frailty measures improve prediction of mortality and morbidity following transcatheter aortic valve implantation? An analysis of the UK TAVI registry
Source: BMJ Open. 2018 Jun 30;8(6):e022543. doi: 10.1136/bmjopen-2018-022543 (PMC6042628; doi:10.1136/bmjopen-2018-022543)
Supplement: Supplementary file 1 [file bmjopen-2018-022543supp001.pdf]

Supplementary Material

Do frailty measures improve prediction of mortality and morbidity following transcatheter aortic valve implantation? An analysis of the UK TAVI registry

Glen P. Martin; Matthew Sperrin; Peter F. Ludman; Mark A. de Belder; Mark Gunning; John Townend; Simon Redwood; Umesh Kadam; Iain Buchan; Mamas A. Mamas

Supplementary Methods

Mathematical Details of Model Updating Techniques

Let P denote the number of predictors that are included in an existing TAVI CPM. Then the jth existing TAVI CPM provides a linear predictor (LP) on the logit scale of the form

$$\text{LP}_{i,j} = \beta_{0,j} + \sum_{p=1}^{P} \beta_{p,j} x_{i,p}$$

for patient i, covariates $x_{i,p}$ for $p \in [1, P]$, and associated coefficients $\beta_{p,j}$.

Then model updating methods, as previously described [1–4], have a hierarchical structure to alter an existing CPM to a new population. This study considered the addition of new variables that were not included in the original TAVI CPMs (i.e. frailty). Specifically, model updating of the jth existing TAVI CPM fits a logistic regression model in the UK TAVI registry with the jth existing LP and a frailty indicator as the only covariates, by modelling

$$\log\left(\frac{\pi_i}{1-\pi_i}\right) = \hat{\alpha}_0 + \hat{\alpha}_1 \text{LP}_{i,j} + \delta_{\text{Frailty}} x_{i,\text{Frailty}} \quad (1)$$

where π_i denotes the predicted event probability for patient i in the UK TAVI registry, $x_{i,\text{Frailty}}$ is either KATZ, CSHA or poor mobility indication, and δ_{Frailty} is the corresponding coefficient estimate. The parameters $\hat{\alpha}_0$ and $\hat{\alpha}_1$ are the calibration intercept and slope, respectively.

This process was repeated for all three of the frailty measures individually and across all of the considered TAVI CPMs. Additionally, to explore the incremental benefit of adding the frailty measures into the TAVI CPMs, we iteratively added each of the frailty measures into Equation (1), under a likelihood ratio test selection criterion until all those frailty measures that significantly improved model fit were selected.

All models were fit in each multiple imputed dataset separately, with the coefficient estimates then pooled according to Rubin's rules. Predictions were then made for each patient in the UK registry, using the pooled coefficient estimates. Subsequent performance estimates (e.g. area under the ROC curve) were made in each imputed dataset, which were then pooled using Rubin's rules.

Supplementary Figures and Tables

Supplementary Figure 1: Distribution of the CSHA frailty score (left panel) and the crude 30-day mortality rate (right panel)

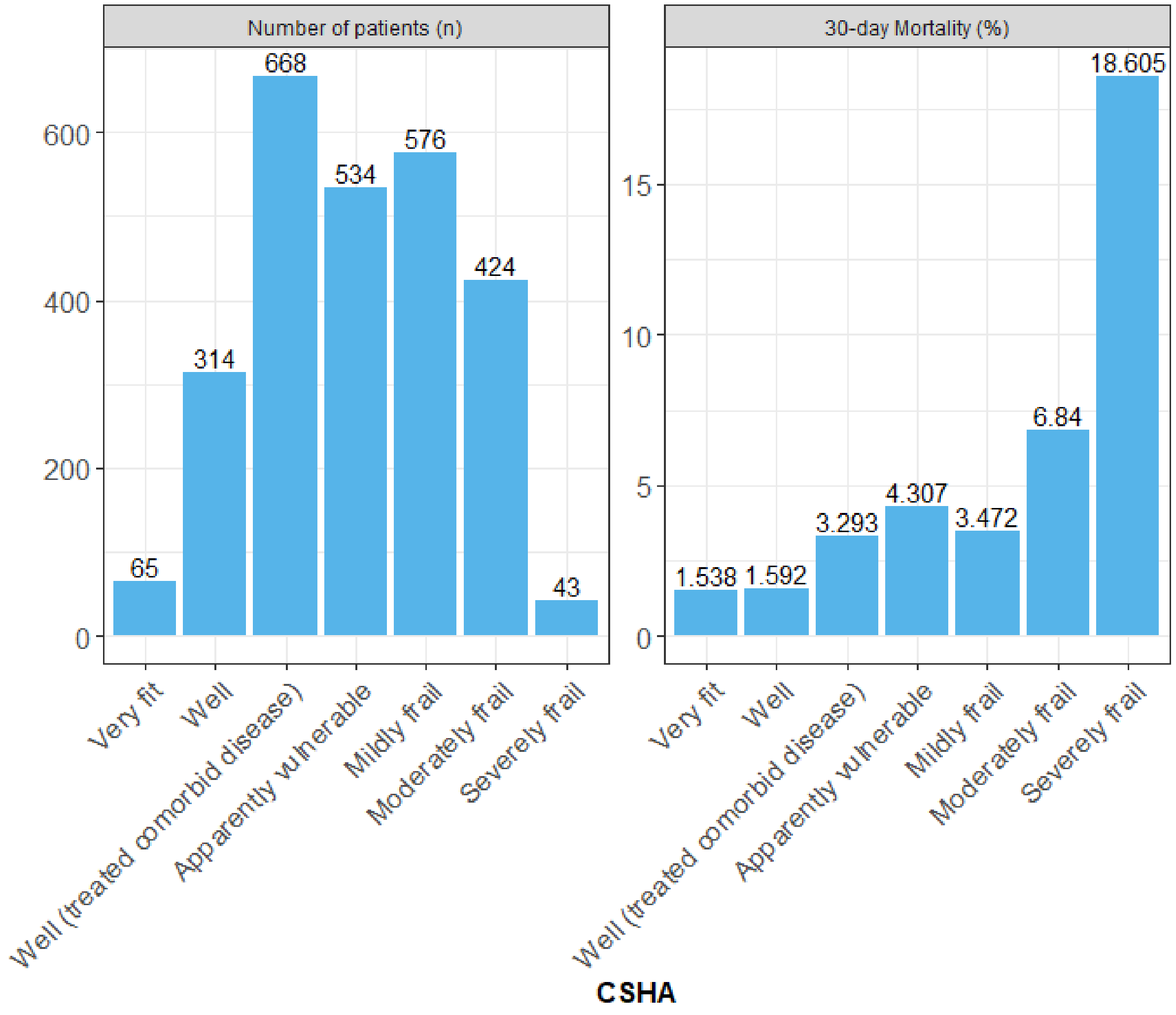

Supplementary Figure 2: Distribution of the KATZ frailty score (left panel) and the crude 30-day mortality rate (right panel)

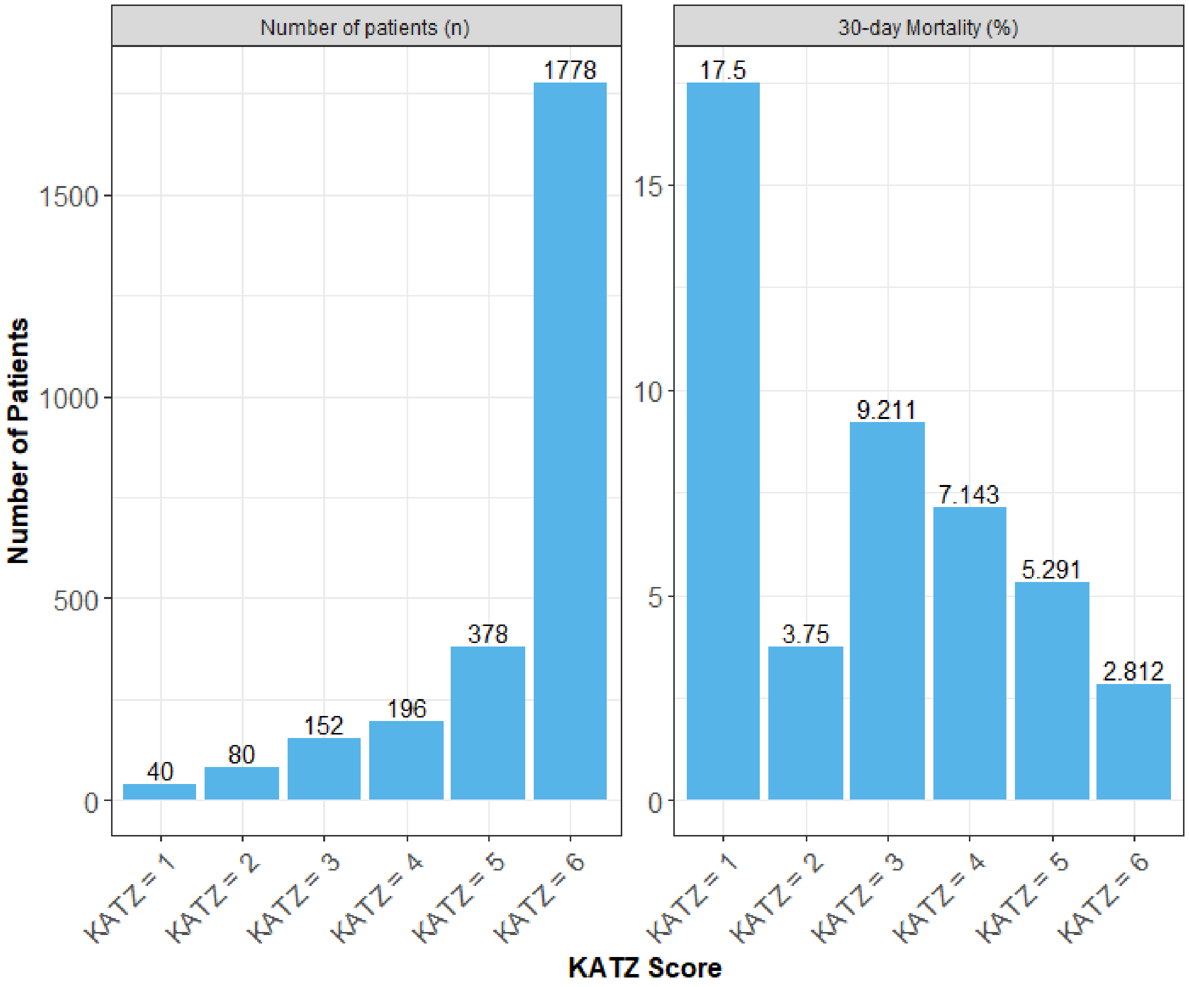

Supplementary Figure 3: Proportion of patients defined as having CSHA-estimated frailty, KATZ activities of daily living dependency, or physician-estimated poor mobility per NHS England region and Wales. The sample size of each region is also depicted.

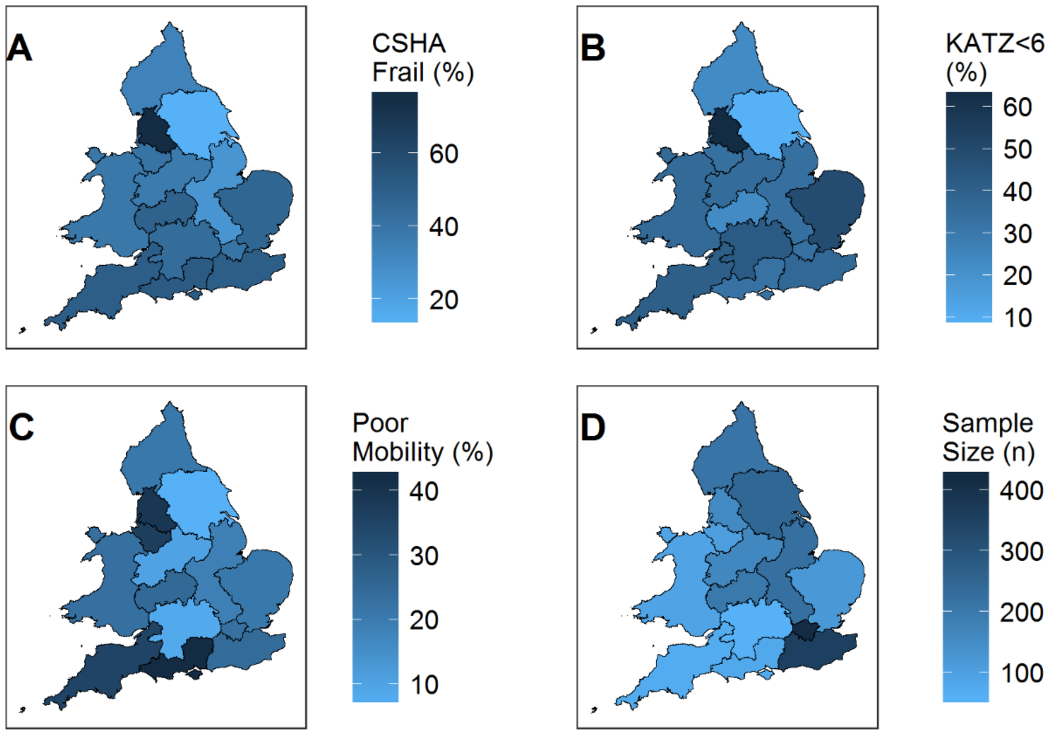

Supplementary Table 1. Baseline characteristics across the patients included and excluded in the analysis.

**Variable**	**Included (n=2624)**	**Excluded (n=449)**	**p-value**
Age, mean (SD)	81.2 (7.58)	80.6 (7.78)	0.127
Female, n (%)	1192 (45.4)	201 (44.8)	0.807
Diabetic, n (%)	641 (24.4)	97 (21.6)	0.573
Smoker, n (%)	1316 (50.2)	193 (43.0)	0.227
Creatinine, mean (SD)	110.9 (60.5)	111.0 (61.2)	0.962
Renal Failure *, n (%)	143 (5.45)	28 (6.24)	0.270
Previous MI, n (%)	558 (21.3)	96 (21.4)	0.506
Pulmonary Disease, n (%)	791 (30.1)	110 (24.5)	0.115
Neurological Disease, n (%)	441 (16.8)	69 (15.4)	0.932
Extracardiac Arteriopathy, n (%)	555 (21.2)	77 (17.1)	0.227
Calcification of Ascending Aorta, % (n)	378 (14.4)	39 (8.69)	0.005
Atrial Fibrillation, n (%)	700 (26.7)	114 (25.4)	0.837
Previous Cardiac Surgery, n (%)	799 (30.4)	109 (24.3)	0.062
Previous PCI, n (%)	524 (20.0)	86 (19.2)	0.903
Height, mean (SD)	1.64 (0.10)	1.66 (0.10)	0.020
Weight, mean (SD)	75.0 (17.1)	76.2 (17.4)	0.209
CCS Class 4, n (%)	24 (0.91)	7 (1.56)	0.243
NYHA ≥ III, n (%)	1985 (75.6)	347 (77.3)	0.001
Aortic Valve Area, mean (SD)	0.69 (0.23)	0.68 (0.23)	0.468
Aortic Valve Peak Gradient, mean (SD)	71.2 (26.2)	72.9 (24.4)	0.243
LVEF < 50%, n (%)	948 (36.1)	161 (35.9)	0.474
One or more diseased vessels, n (%)	1058 (40.3)	151 (33.6)	0.064
Left Main Stem Disease, n (%)	103 (3.93)	18 (4.01)	0.866
Non-elective Procedure, n (%)	365 (13.9)	62 (13.8)	0.999
Access Site			
Transfemoral, n (%)	2127 (81.1)	334 (74.4)	0.005
Transapical, n (%)	249 (9.49)	75 (16.7)	<0.001
Subclavian, n (%)	85 (3.24)	5 (1.11)	0.022
Other, n (%)	160 (6.10)	30 (6.68)	0.674

** Defined as creatinine >200µmol/l or dialysis for renal failure. LVEF: Left Ventricular Ejection Fraction, MI: Myocardial Infarction, NYHA: New York Heart Association, PCI: Percutaneous coronary intervention.*

Supplementary Table 2. Baseline characteristics across CSHA-estimated frailty groups.

**Variable**	**CSHA-estimated Frail (n=1043)**	**CSHA-estimated Non-Frail (n=1581)**	**p-value**
Age, mean (SD)	82.0 (7.13)	80.7 (7.82)	<0.001
Female, n (%)	525 (50.3)	667 (42.2)	<0.001
Diabetic, n (%)	280 (26.8)	361 (22.8)	0.021
Smoker, n (%)	518 (49.7)	798 (50.5)	0.962
Creatinine, mean (SD)	116.5 (67.0)	107.2 (55.6)	<0.001
Renal Failure *, n (%)	72 (6.90)	71 (4.49)	0.010
Previous MI, n (%)	246 (23.6)	312 (19.7)	0.021
Pulmonary Disease, n (%)	321 (30.8)	470 (29.7)	0.564
Neurological Disease, n (%)	199 (19.1)	242 (15.3)	0.014
Extracardiac Arteriopathy, n (%)	268 (25.7)	287 (18.2)	<0.001
Calcification of Ascending Aorta, % (n)	116 (11.1)	262 (16.6)	<0.001
Atrial Fibrillation, n (%)	296 (28.4)	404 (25.6)	0.138
Previous Cardiac Surgery, n (%)	276 (26.5)	523 (33.1)	<0.001
Previous PCI, n (%)	201 (19.3)	323 (20.4)	0.501
Height, mean (SD)	1.63 (0.10)	1.65 (0.10)	<0.001
Weight, mean (SD)	73.7 (17.5)	75.8 (16.7)	0.003
CCS Class 4, n (%)	12 (1.15)	12 (0.76)	0.412
NYHA ≥ III, n (%)	922 (88.4)	1063 (67.2)	<0.001
Aortic Valve Area, mean (SD)	0.68 (0.22)	0.69 (0.24)	0.092
Aortic Valve Peak Gradient, mean (SD)	67.4 (26.2)	73.7 (25.9)	<0.001
LVEF < 50%, n (%)	416 (39.9)	532 (33.6)	0.001
One or more diseased vessels, n (%)	439 (42.1)	619 (39.2)	0.190
Left Main Stem Disease, n (%)	56 (5.37)	47 (2.97)	0.002
Non-elective Procedure, n (%)	199 (19.1)	166 (10.5)	<0.001
Access Site			
Transfemoral, n (%)	840 (80.5)	1287 (81.4)	0.546
Transapical, n (%)	91 (8.72)	158 (9.99)	0.302
Subclavian, n (%)	34 (3.26)	51 (3.23)	0.999
Other, n (%)	78 (7.48)	82 (5.19)	0.021

** Defined as creatinine >200µmol/l or dialysis for renal failure. LVEF: Left Ventricular Ejection Fraction, MI: Myocardial Infarction, NYHA: New York Heart Association, PCI: Percutaneous coronary intervention.*

Supplementary Table 3. Baseline characteristics across frailty groups defined with KATZ<6.

**Variable**	**KATZ<6 (n=846)**	**KATZ=6 (n=1778)**	**p-value**
Age, mean (SD)	81.5 (7.38)	81.1 (7.67)	0.199
Female, n (%)	417 (49.3)	775 (43.6)	0.007
Diabetic, n (%)	217 (25.7)	424 (23.8)	0.347
Smoker, n (%)	434 (51.3)	882 (49.6)	0.252
Creatinine, mean (SD)	113.6 (69.1)	109.6 (56.0)	0.121
Renal Failure *, n (%)	48 (5.67)	95 (5.34)	0.783
Previous MI, n (%)	198 (23.4)	360 (20.2)	0.075
Pulmonary Disease, n (%)	279 (33.0)	512 (28.8)	0.028
Neurological Disease, n (%)	159 (18.8)	282 (15.9)	0.069
Extracardiac Arteriopathy, n (%)	184 (21.7)	371 (20.9)	0.635
Calcification of Ascending Aorta, % (n)	134 (15.8)	244 (13.7)	0.167
Atrial Fibrillation, n (%)	263 (31.1)	437 (24.6)	<0.001
Previous Cardiac Surgery, n (%)	228 (27.0)	571 (32.1)	0.009
Previous PCI, n (%)	165 (19.5)	359 (20.2)	0.725
Height, mean (SD)	1.64 (0.10)	1.65 (0.10)	0.227
Weight, mean (SD)	75.1 (17.0)	74.9 (17.1)	0.843
CCS Class 4, n (%)	10 (1.18)	14 (0.79)	0.439
NYHA ≥ III, n (%)	677 (80.0)	1308 (73.6)	<0.001
Aortic Valve Area, mean (SD)	0.68 (0.23)	0.69 (0.23)	0.536
Aortic Valve Peak Gradient, mean (SD)	70.4 (24.4)	71.6 (27.1)	0.271
LVEF < 50%, n (%)	356 (42.1)	592 (33.3)	<0.001
One or more diseased vessels, n (%)	352 (41.6)	706 (39.7)	0.416
Left Main Stem Disease, n (%)	36 (4.26)	67 (3.77)	0.655
Non-elective Procedure, n (%)	161 (19.0)	204 (11.5)	<0.001
Access Site			
Transfemoral, n (%)	704 (83.2)	1423 (80.0)	0.070
Transapical, n (%)	76 (8.98)	173 (9.73)	0.581
Subclavian, n (%)	22 (2.60)	63 (3.54)	0.244
Other, n (%)	44 (5.20)	116 (6.52)	0.213

** Defined as creatinine >200µmol/l or dialysis for renal failure. LVEF: Left Ventricular Ejection Fraction, MI: Myocardial Infarction, NYHA: New York Heart Association, PCI: Percutaneous coronary intervention.*

Supplementary Table 4. Baseline characteristics across those with/without physician-estimated poor mobility.

Variable	Poor Mobility (n=591)	Normal Mobility (n=2033)	p-value
Age, mean (SD)	81.4 (7.92)	81.1 (7.47)	0.481
Female, n (%)	328 (55.5)	864 (42.5)	<0.001
Diabetic, n (%)	151 (25.5)	490 (24.1)	0.496
Smoker, n (%)	288 (48.7)	1028 (50.6)	0.677
Creatinine, mean (SD)	115.3 (80.3)	109.6 (53.4)	0.045
Renal Failure *, n (%)	42 (7.11)	101 (4.97)	0.055
Previous MI, n (%)	128 (21.7)	430 (21.2)	0.844
Pulmonary Disease, n (%)	206 (34.9)	585 (28.8)	0.004
Neurological Disease, n (%)	112 (19.0)	329 (16.2)	0.129
Extracardiac Arteriopathy, n (%)	165 (27.9)	390 (19.2)	<0.001
Calcification of Ascending Aorta, % (n)	91 (15.4)	287 (14.1)	0.371
Atrial Fibrillation, n (%)	168 (28.4)	532 (26.2)	0.287
Previous Cardiac Surgery, n (%)	131 (22.2)	668 (32.9)	<0.001
Previous PCI, n (%)	93 (15.7)	431 (21.2)	0.004
Height, mean (SD)	1.63 (0.10)	1.65 (0.10)	<0.001
Weight, mean (SD)	74.8 (17.9)	75.0 (16.8)	0.818
CCS Class 4, n (%)	7 (1.18)	17 (0.84)	0.590
NYHA ≥ III, n (%)	489 (82.7)	1496 (73.6)	<0.001
Aortic Valve Area, mean (SD)	0.67 (0.23)	0.69 (0.23)	0.110
Aortic Valve Peak Gradient, mean (SD)	69.3 (25.3)	71.8 (26.5)	0.050
LVEF < 50%, n (%)	224 (37.9)	724 (35.6)	0.308
One or more diseased vessels, n (%)	231 (39.1)	827 (40.7)	0.551
Left Main Stem Disease, n (%)	18 (3.05)	85 (4.18)	0.282
Non-elective Procedure, n (%)	120 (20.3)	245 (12.1)	<0.001
Access Site			
Transfemoral, n (%)	454 (76.8)	1673 (82.3)	0.004
Transapical, n (%)	80 (13.5)	169 (8.31)	<0.001
Subclavian, n (%)	23 (3.89)	62 (3.05)	0.374
Other, n (%)	33 (5.58)	127 (6.25)	0.623

** Defined as creatinine >200µmol/l or dialysis for renal failure. LVEF: Left Ventricular Ejection Fraction, MI: Myocardial Infarction, NYHA: New York Heart Association, PCI: Percutaneous coronary intervention.*

Supplementary Table 5. Multivariable adjusted **s**hort-term outcomes for the sensitivity analysis of the composite frailty indicator. Bold items indicate significant results.

**Composite Frailty**	**Moderately frail vs. non-frail multivariable OR (95% CI) ***	**Severely frail vs. non-frail multivariable OR (95% CI) ***
30-day mortality	1.19 (0.74, 1.92)	**2.99 (1.71, 5.23)**
Early Safety	1.18 (0.91, 1.52)	**1.88 (1.32, 2.68)**

**: Severely Frail (N=297) corresponds to a patient defined as frail across all three of CSHA, KATZ and poor mobility; Moderately Frail (N=1169) corresponds to a patient that is defined frail by at least one (but not all) of CSHA, KATZ and poor mobility; Non-frail (N=1158) means a patient was defined as not frail by all of CSHA, KATZ and poor mobility.*

CI: Confidence Interval, MVC: Major Vascular Complication, OR: Odds Ratio, PPM: Permanent Pacemaker Implantation

Supplementary References

1 Janssen KJM, Moons KGM, Kalkman CJ, *et al.* Updating methods improved the performance of a clinical prediction model in new patients. *J Clin Epidemiol* 2008;**61**:76–86. doi:10.1016/j.jclinepi.2007.04.018

2 Steyerberg EW, Borsboom GJJM, van Houwelingen HC, *et al.* Validation and updating of predictive logistic regression models: a study on sample size and shrinkage. *Stat Med* 2004;**23**:2567–86. doi:10.1002/sim.1844

3 Moons KGM, Kengne AP, Grobbee DE, *et al.* Risk prediction models: II. External validation, model updating, and impact assessment. *Heart* 2012;**98**:691–8. doi:10.1136/heartjnl-2011-301247

4 Su T-L, Jaki T, Hickey GL, *et al.* A review of statistical updating methods for clinical prediction models. *Stat Methods Med Res* Published Online First: 26 July 2016. doi:10.1177/0962280215626466
