# Supplementary material for: Do frailty measures improve prediction of mortality and morbidity following transcatheter aortic valve implantation? An analysis of the UK TAVI registry
Source: BMJ Open. 2018 Jun 30;8(6):e022543. doi: 10.1136/bmjopen-2018-022543 (PMC6042628; doi:10.1136/bmjopen-2018-022543)
Supplement: Author's manuscript [file bmjopen-2018-022543.draft_revisions.pdf]

BMJ Open is committed to open peer review. As part of this commitment we make the peer review history of every article we publish publicly available.

When an article is published we post the peer reviewers' comments and the authors' responses online. We also post the versions of the paper that were used during peer review. These are the versions that the peer review comments apply to.

The versions of the paper that follow are the versions that were submitted during the peer review process. They are not the versions of record or the final published versions. They should not be cited or distributed as the published version of this manuscript.

BMJ Open is an open access journal and the full, final, typeset and author-corrected version of record of the manuscript is available on our site with no access controls, subscription charges or pay-per-view fees (http://bmjopen.bmj.com).

If you have any questions on BMJ Open's open peer review process please email info.bmjopen@bmj.com

BMJ Open

Frailty measures are key to better prediction of mortality and morbidity following transcatheter aortic valve implantation: an analysis of the UK TAVI registry

Journal:	*BMJ Open*
Manuscript ID	bmjopen-2018-022543
Article Type:	Research
Date Submitted by the Author:	22-Feb-2018
Complete List of Authors:	Martin, Glen; University of Manchester Institute of Population Health, Farr Institute<br>Sperrin, Matthew; University of Manchester Institute of Population Health, Farr Institute<br>Ludman, Peter; University Hospitals Birmingham NHS Foundation Trust, Cardiology<br>deBelder, Mark; South Tees NHS Foundation Trust, Cardiology<br>Gunning, Mark ; Keele University, Keele Cardiovascular Research Group<br>Townend, Jonathan; University Hospitals Birmingham NHS Foundation Trust, Cardiology<br>Redwood, Simon R; St Thomass Hospital<br>Kadam, Umesh; Keele University, Keele Cardiovascular Research Group<br>Buchan, Iain; Microsoft Research<br>Mamas, Mamas; Keele University, Keele Cardiovascular Research Group
Keywords:	Frailty, Transcatheter Aortic Valve Implantation, Mortality, Risk Stratification, Clinical Prediction Models

SCHOLARONE™
Manuscripts

Clinical Investigation

Frailty measures are key to better prediction of mortality and morbidity following transcatheter aortic valve implantation: an analysis of the UK TAVI registry

Short title: Frailty and outcomes following TAVI

Glen P. Martin, PhD [1]; Matthew Sperrin, PhD [1]; Peter F. Ludman, MA, MD, FRCP, FESC [2]; Mark A. de Belder, MA, MD, FRCP [3]; Mark Gunning, MD, FRCP [4]; John Townend, MD [2]; Simon Redwood, MBBS, MD [5]; Umesh Kadam, MD [4]; Iain Buchan, MD, FFPH [1,6]; Mamas A. Mamas, MA, DPhil, FRCP [1,4]

1: Farr Institute, Faculty of Biology, Medicine and Health, University of Manchester, Manchester Academic Health Science Centre, Manchester, United Kingdom

2: Queen Elizabeth Hospital, Birmingham, United Kingdom

3: The James Cook University Hospital, Middlesbrough, United Kingdom

4: Keele Cardiovascular Research Group, Institute of Applied Clinical Science and Centre for Prognosis Research Group, Institute of Primary Care and Health Sciences, Keele University, and Academic Department of Cardiology, Royal Stoke Hospital, Stoke-on-Trent, UK

5: Guys & St Thomas' NHS Foundation Trust, London, UK

6: Microsoft Research, Cambridge, UK

Manuscript Word Count: 3571

Corresponding author:

Mamas A. Mamas

Professor of Cardiology / Honorary Consultant Cardiologist

Keele Cardiovascular Research Group, Guy Hilton Research Centre, Thornburrow Drive, Keele University, Stoke-on-Trent, ST4 7QB, UK

Email: mamasmamas1@yahoo.co.uk

For peer review only - http://bmjopen.bmj.com/site/about/guidelines.xhtml

Structured Abstract

Objectives

Preliminary studies indicate frailty to be associated with poor outcomes following transcatheter aortic valve implantation (TAVI), but there is limited evidence from multicentre registries. The aim was to investigate the independent association of frailty with TAVI outcomes, and the prognostic utility of adding frailty into existing clinical prediction models (CPMs).

Design

The UK TAVI registry incorporated three frailty measures since 2013: CSHA, KATZ and poor mobility. We investigated the associations between these frailty measures with short- and long-term outcomes, using logistic regression to estimate multivariable adjusted odds ratios (ORs), and Cox proportional hazards models to explore long-term survival. We compared the predictive performance of existing TAVI CPMs before and after updating them to include each frailty measure.

Setting

All patients who underwent a TAVI procedure in England or Wales between 2013 and 2014.

Participants

2624 TAVI procedures were analysed in this study.

Primary and Secondary Outcomes

The primary endpoints in this study were 30-day mortality and long-term survival. The Valve Academic Research Consortium (VARC)-2 composite early safety endpoint was considered as a secondary outcome.

For peer review only - http://bmjopen.bmj.com/site/about/guidelines.xhtml

Results

KATZ<6 (OR 2.10, 95% CI: 1.39, 3.15) and poor mobility (OR 2.15, 95% CI: 1.41, 3.28) predicted 30-day mortality after multivariable adjustment. All frailty measures were associated with increased odds of the Valve Academic Research Consortium (VARC)-2 composite early safety endpoint. We observed a significant increase in the area under the ROC curves by approximately 5% after adding KATZ<6 or poor mobility. Risk stratification agreement was significantly improved by the addition of each frailty measure, with an increase in intra-class correlation coefficient of between 0.15 and 0.31.

Conclusion

Frailty was associated with worse outcomes following TAVI, and incorporating frailty metrics significantly improved the predictive performance of existing CPMs. Physician-estimated frailty measures could aid TAVI risk stratification, until more objective scales are routinely collected.

Keywords

Frailty – Transcatheter Aortic Valve Implantation – Mortality – Risk Stratification – Clinical Prediction Models

For peer review only

Article Summary

Strengths and Limitations of this Study

- An analysis of the association between three frailty measures and clinical outcomes following transcatheter aortic valve implantation (TAVI).
- The study used data from the UK TAVI registry, including all consecutive patients who underwent TAVI in UK between 2013 and 2014.
- A large, contemporary study, which investigated the impact of frailty on outcomes and mortality prediction post TAVI from a national perspective.
- Only three frailty measures were available, with objective frailty tests not recorded.
- This retrospective analysis could not compare outcomes across frailty groups in untreated aortic stenosis patients, or those undergoing surgical replacement.

For peer review only

Introduction

Transcatheter aortic valve implantation (TAVI) has emerged as an effective treatment strategy for patients with aortic stenosis who are intermediate-to-high operative risk [1–3]. Nevertheless, successful outcomes following TAVI depend on careful patient selection. Current guidelines advocate the procedure in high-risk patients, based on heart team assessment in combination with multiple clinical prediction models (CPMs) [4]. To this end, surgical CPMs, such as the EuroSCORE or the STS model, have previously been used, but TAVI specific CPMs are emerging from national registries: examples include the FRANCE-2 model [5], the OBSERVANT model [6] and the American College of Cardiology Transcatheter Valve Therapy (ACC) model [7]. However, these models do not usually incorporate measures of frailty and disability into geriatric pre-screening risk assessment.

Frailty is an emerging concept in clinical science and is defined as an age-related decline in the resilience to stressors caused by deterioration in multiple physiological systems [8]. Previous studies have indicated associations between frailty and poor outcomes after cardiac surgery [9,10], with emerging evidence of similar associations following TAVI [11–15]. Patient's frailty, dependencies in activities of daily living (ADL) and cognitive function are increasingly being considered in the decision-making process for TAVI eligibility [16], but such measures are not incorporated into many of the currently available CPMs, which has potentially contributed to the moderate predictive performance of such models outside their development datasets [17–19]. The recent recording of measures relating to frailty/disability in national cohorts presents novel opportunities to incorporate them into future iterations of existing TAVI CPMs.

Therefore, the aim was to investigate the effect of frailty and disability on mortality and morbidity in a national TAVI cohort and to examine the prognostic utility of adding frailty/disability into existing TAVI CPMs.

For peer review only - http://bmjopen.bmj.com/site/about/guidelines.xhtml

Methods

UK TAVI Registry

The UK TAVI registry prospectively collects 95 variables (including patient demographics, risk factors for intervention and within-hospital adverse outcomes), for every TAVI procedure conducted in the UK [20]. This analysis used data from patients in England and Wales, for whom all-cause mortality was linked from the Office for National Statistics. The registry recorded three variables that related to frailty/disability from 2013, and so the study period for the analysis was January 2013 to December 2014. Specifically, the registry recorded the Canadian Study of Health and Aging (CSHA)-estimated frailty scale [21], KATZ ADL dependency [22], and a physician-estimated poor mobility. Poor mobility was defined as per the EuroSCORE II model as any impairment of mobility that is secondary to musculoskeletal or neurological dysfunction [23]. Frailty/disability groups were defined separately across the three measures with the full definitions of each group given in **Table 1**. Dichotomising CSHA and KATZ scores into two categories was based on the empirical median value (CSHA="apparently vulnerable" and KATZ=6), and on the original publication of CSHA [21]. Additionally, for this analysis, we defined a composite score to incorporate information from all three of the measures, with the definition given in **Table 1**.

Study Endpoints

The primary endpoints in this study were 30-day mortality and long-term survival. As a secondary endpoint, we investigated the Valve Academic Research Consortium (VARC)-2 composite early safety endpoint, which is defined as a failure in any of the following outcomes by 30-days: all-cause mortality, stroke, life-threatening bleeding, stage 2/3 acute kidney injury, coronary artery obstruction requiring intervention, major vascular complications, or valve-related dysfunction requiring a repeat procedure [16]. The

For peer review only - http://bmjopen.bmj.com/site/about/guidelines.xhtml

components of composite early safety were not analysed individually due to low event numbers, meaning any such analysis would be underpowered.

Statistical Analysis

Every variable with missing data was imputed using multiple imputation that generated ten imputed datasets [24]. The imputation model for each variable included the majority of other variables in the UK TAVI registry and all considered endpoints [25]. Imputed frailty/disability measures and outcomes were not used, and instead were returned to the original (missing) values [26]. Subsequently, patients missing data on CSHA, KATZ, poor mobility, and/or missing life status were removed from the analysis; patients with missing VARC-2 composite early safety were only removed from the analysis of that outcome. All analyses were undertaken in each imputed dataset separately, with the results pooled according to Rubin's rules [24].

For exploratory analysis, we defined a centre-level volume indication for the study period (number of procedures conducted per centre between January 2013 and December 2014). Centre volume was stratified per 50 procedural increase; within each volume strata, we calculated the proportion of frail patients defined across each of the three measures. Additionally, we obtained spatial maps of England at a National Health Service (NHS) regional level (a high-level geographical structure partitioning England into 13 regions) and of Wales, from the Office for National Statistics. By linking the clinical commission group (local area NHS bodies responsible for planning and commissioning of health care) of each patient to the corresponding NHS region, we calculated the proportion of frail patients in the study population across England and Wales.

Odds ratios (ORs) for binary endpoints were estimated using logistic regression, with both unadjusted and adjusted ORs reported. Multivariable models adjusted for the following variables, which included the majority of variables that are recorded in the registry and are

For peer review only - http://bmjopen.bmj.com/site/about/guidelines.xhtml

available prior or during the procedure: total centre volume, age, sex, diabetes status, smoking status, creatinine, renal failure, previous myocardial infarction, pulmonary disease, neurological disease, extra-cardiac arteriopathy, calcification of ascending aorta, atrial fibrillation/flutter, previous cardiac surgery, previous percutaneous coronary intervention (PCI), height, weight, critical pre-operative status, Canadian Cardiovascular Society grading class 4, New York Heart Association (NYHA) class ≥III, aortic valve area and peak gradient, Left Ventricular Ejection Fraction (LVEF) <50%, one or more diseases vessels, left main stem disease, non-elective procedure indication and non-transfemoral access indication.

Long-term survival was assessed non-parametrically using Kaplan-Meier plots and strata were compared using the log-rank test. Multivariable adjustment was performed using Cox proportional hazards models, using the same variables as above to adjust the frailty indication hazard ratio (HR) for measured confounding. To account for non-proportional hazards, time was split into strata of 0-180 days, 180-365 days and >365 days, with frailty-indication-by-time-indicator interactions included in the Cox proportional hazards models.

The effect on predictive performance of adding CSHA-estimated frailty, KATZ ADL dependency, or physician-estimated poor mobility into the existing TAVI CPMs (FRANCE-2, OBSERVANT and ACC) was considered using model updating techniques [27–29]; **Supplementary Table 1** gives the variables included in the original versions of these models. Mathematical details of model updating techniques are given in the supplementary methods. In short, CSHA, KATZ, and poor mobility were added (both separately for each measure, and sequentially through forward stepwise selection) into an existing CPM by fitting a logistic regression model of 30-day mortality, with both the linear predictor of the considered TAVI CPM and at least one of the frailty/disability measures as covariates. The likelihood ratio test (LRT) was used to test for significant improvement in model fit between the original version of each TAVI CPM and the frailty-updated versions. Additionally, the area under the receiver

For peer review only - http://bmjopen.bmj.com/site/about/guidelines.xhtml

operating characteristic curve (AUC) was calculated for each model and compared with the original model using a DeLong comparison [30]. Finally, intra-class correlation coefficient (ICC) estimates and their 95% CI were calculated based on an absolute-agreement, two-way random-effects model to indicate the risk-prediction agreement between the TAVI CPMs both before and after the addition of CSHA, KATZ, or poor mobility.

R version 3.4.0 [31] was used for all statistical analyses. Graphical plots were made using the ggplot2 package [32] and the mice package was used for the multiple imputation [33].

Results

Between January 2013 and December 2014, n=3073 patients underwent a TAVI procedure in England and Wales. After excluding 104 patients with missing life-status and 345 patients with any missing frailty measure, 2624 patients were analysed in this study. Due to the relative high proportion of exclusions (14.6%), we examined baseline characteristics of included patients compared with those excluded (**Supplementary Table 2**). Most of the baseline characteristics were similar between the study group and the excluded group, with the exception of access route, proportion of calcified aorta and NYHA≥ III.

A total of 1043 patients (39.7%) had CSHA-estimated frailty (**Supplementary Figure 1**), 846 patients (32.2%) had ADL dependency (KATZ<6) (**Supplementary Figure 2**), and 591 patients (22.5%) had physician-estimated poor mobility. **Figure 1** shows a Venn diagram of the overlap in each frailty/disability classification across the three measures. The central segment of the Venn diagram (n=297) is the group defined as “Severely Frail” by the composite score, with the other segments (n=1169) showing the “Moderately Frail” group.

Baseline characteristics for the whole cohort and across CSHA-estimated frailty groups are given in **Table 2**, with similar comparisons made between KATZ ADL

For peer review only - http://bmjopen.bmj.com/site/about/guidelines.xhtml

dependency groups (**Table 3**) and between physician-estimated poor mobility groups (**Table 4**). Patients were significantly older when defined as CSHA-estimated frail ($p<0.001$), but not by KATZ ADL dependency ($p=0.199$) or physician-estimated poor mobility ($p=0.481$). CSHA-estimated frail patients were more likely female ($p<0.001$), with higher proportions of NYHA≥III ($p<0.001$), LVEF<50% ($p=0.001$) and non-elective procedures ($p<0.001$), but with lower numbers of CSHA-estimated frail patients having a previous cardiac surgery ($p<0.001$); similar differences were observed between KATZ ADL dependency and physician-estimated poor mobility groups. The prevalence of extra cardiac arteriopathy was higher in CSHA-estimated frailty ($p<0.001$) and physician-estimated poor mobility ($p<0.001$) groups, and there were higher proportions of patients with pulmonary disease in those with physician-estimated poor mobility ($p=0.004$) or KATZ<6 ($p=0.028$). Patients with physician-estimated poor mobility underwent transfemoral access less than those with normal mobility ($p=0.004$).

Generally, the proportion of patients defined as "Non-Frail" by the composite score increased with higher total centre volume (**Figure 2**). Specifically, the proportion of patients who the physician estimated as having poor mobility was lower in centres with higher total centre volume ($p<0.001$), and proportions of KATZ<6 was observed significantly less in centres with over 100 procedures ($p<0.001$) (**Supplementary Figure 3**). In contrast, proportions of patients with CSHA-estimated frailty increased with increasing total centre volume ($p=0.042$) (**Supplementary Figure 3)**.

There was large spatial heterogeneity in the proportion of TAVI procedures conducted in patients estimated as having poor mobility, KATZ<6 or CSHA-estimated frailty (**Supplementary Figure 4**). For example, proportions of physician-estimated poor mobility patients across NHS regions ranged from 6.5% to 42.7%; NHS England North (Lancashire and Greater Manchester) had the highest proportion of patients with KATZ<6 and CSHA-

For peer review only - http://bmjopen.bmj.com/site/about/guidelines.xhtml

estimated frailty, while NHS England South (Wessex) had the highest proportion of physician-estimated poor mobility patients.

Frailty and Post TAVI Outcomes

Incremental increases in components of CSHA and KATZ were associated with increased crude 30-day mortality rates (**Supplementary Figure 1** and **Supplementary Figure 2**). Specifically, CSHA-estimated frail patients had higher crude 30-day mortality (OR 1.73, 95% CI: 1.18, 2.55), but this was not significant after multivariable adjustment (**Table 5**). In contrast, physician-estimated poor mobility (OR 2.15, 95% CI: 1.41, 3.28) and KATZ<6 (OR 2.10, 95% CI: 1.39, 3.15) were significantly associated with increased odds of 30-day mortality after multivariable adjustment. All three of the considered frailty/disability measures were independently associated with increased VARC-2 composite early safety endpoint (**Table 5**). Similarly, those defined as frail across all three of CSHA, KATZ and poor mobility (i.e. Severely Frail within the composite score) had significantly higher multivariable adjusted odds of 30-day mortality and composite early safety compared with patients defined as Non-Frail by the composite score (**Supplementary Table 3**).

Overall survival was 95.9%, 85.2% and 76.3% at 30-days, one-year and two-years, respectively. Observed survival was significantly worse for patients with CSHA-estimated frailty ($p<0.0001$), KATZ<6 ($p<0.0001$), physician-estimated poor mobility ($p<0.001$), and across the composite frailty groups ($p<0.0001$) (**Figure 3)**. The multivariable time-dependent Cox proportional hazards models indicated that hazards of mortality were significantly higher for those with KATZ<6 or physician-estimated poor mobility within one-year post TAVI (**Table 6**); after one-year, multivariable adjusted hazards were not significantly different for these measures. CSHA-estimated frailty had significantly increased hazards of mortality 0-180 days and >365 days post TAVI, but hazards were not significantly different between

For peer review only - http://bmjopen.bmj.com/site/about/guidelines.xhtml

these time windows (HR 1.37, 95% CI: 0.94, 2.01). Quantitatively similar results were found across the composite score frailty groups.

Addition of Frailty in TAVI CPMs

Table 7 gives the discrimination of each TAVI CPM for predicting 30-day mortality both before and after the addition of each frailty/disability measure. The LRT indicated a significant improvement in model fit by adding CSHA-estimated frailty into the FRANCE-2 model (p=0.037), the OBSERVANT model (p=0.020) and the ACC model (p=0.048). Adding KATZ<6 into the TAVI models significantly improved the AUC of both the FRANCE-2 CPM (p=0.047) and the OBSERVANT CPM (p=0.007), with the LRT indicating a significant improvement in model fit for all three TAVI CPMs (p<0.001). Equally, physician-estimated poor mobility significantly improved the fit of all three models (LRT p<0.001) and significantly increased the AUC of the OBSERVANT (p=0.006) and the ACC (p=0.030) CPMs by 7% and 5%, respectively. A forward stepwise selection of all three measures resulted in KATZ<6 and physician-estimated poor mobility being added into each of the existing TAVI CPMs (**Table 7**); that is, CSHA-estimated frailty did not significantly improve the predictive performance of the existing TAVI models after inclusion of KATZ<6 and physician-estimated poor mobility.

Finally, the absolute-agreement ICC between the original versions of each TAVI CPM was 0.39 (95% CI: 0.27, 0.49), which increased to 0.65 (95% CI: 0.63, 0.67), 0.78 (95% CI: 0.76, 0.79) and 0.81 (95% CI: 0.79, 0.82) upon adding CSHA-estimated frailty, KATZ<6 or physician-estimated poor mobility, respectively. Thus, the patient-level agreement in risk-prediction between the three TAVI CPMs was significantly improved after adding any of CSHA, KATZ, or poor mobility into the models.

For peer review only - http://bmjopen.bmj.com/site/about/guidelines.xhtml

Discussion

The findings from this study can be summarised as follows: 1) KATZ<6 and physician-estimated poor mobility were independently associated with increased 30-day mortality, and all three considered frailty/disability measures were associated with increased VARC-2 composite early safety; 2) patients with KATZ<6 and physician-estimated poor mortality had significantly worse survival up to one-year post TAVI, while CSHA-estimated frailty was associated with increased mortality hazards 0-180 days and >365 days post TAVI; 3) the addition of KATZ and physician-estimated poor mobility into existing TAVI CPMs significantly improved the discrimination and patient-level risk-stratification agreement of the models.

Given that frailty is an indicator of general health status and vulnerability among elderly patients [8], it is naturally under study in the TAVI field. The current study supports and expands the findings from previous studies that have shown associations between frailty and poor TAVI outcomes [11–15,34,35]. For instance, a previous analysis of a single UK centre showed that poor mobility strongly predicted survival following TAVI [13]; the current study supports this finding at the national level, in showing that the odds of 30-day mortality in those with physician-estimated poor mobility were 2.15 times higher compared with normal mobility. Similarly, a multicentre investigation of the Japanese registry found that a semi-quantitative clinical frailty scale was associated with increased 30-day and one-year mortality [15]. Together, the current and previous results suggest that frailty/disability is an indicator of worse outcomes post TAVI, despite the heterogeneity in frailty measures used across the existing evidence base. AHA/ACC guidelines currently indicate that KATZ, gait speed and grip strength should be used in the assessment of surgical risk [36], with the emerging data suggesting that similar recommendations should be made for TAVI risk assessment and prediction.

For peer review only - http://bmjopen.bmj.com/site/about/guidelines.xhtml

However, implementing this emerging evidence may prove difficult without consensus regarding the most clinically useful measure of frailty in TAVI patients. By comparing the predictive performance of 7 different frailty scales, Afilalo et al. recently recommended that the "Essential Frailty Toolset", which includes multiple domains of frailty (motor skills, cognition, and nutritional/physiological factors), be used in TAVI patients [14]. Data on such frailty-domains were unavailable for the current study, with the three measures collected within the UK TAVI registry mainly utilising measurements of ADL. Nonetheless, it is conceivable that assessment of ADL (e.g. KATZ) could provide a simple mechanism of informing part of the risk assessment process for TAVI, until more objective frailty scales are routinely collected. For instance, a previous multicentre study by Alfredsson et al. showed that an objective measure of gait speed was associated with 30-day mortality following TAVI [34], but such measures are rarely recorded in national registries, which would currently limit their use to aid risk prediction/stratification in TAVI patients.

Prognostic prediction for TAVI is an ongoing research area, with existing TAVI CPMs reporting only moderate performance when validated [5–7,18]. We found that adding indicators of dependency in ADL (KATZ<6) or physician-estimated poor mobility significantly improved the predictive performance and patient-level risk-stratification agreement of the TAVI models. This supports previous work that demonstrated improvements in predictive performance of the EuroSCORE and STS models through the addition of frailty measures [14,15]. Such results have important clinical implications from a risk stratification perspective. In particular, the heart team should consider frailty measures in addition to the multiple comorbidities that are reflected in existing risk scores, preferably by the inclusion of frailty directly into the risk prediction. We recommend that future iterations of existing and new TAVI models should include markers of frailty/disability. Interestingly, CSHA-estimated frailty was not independently associated with 30-day mortality and did not

For peer review only - http://bmjopen.bmj.com/site/about/guidelines.xhtml

significantly improve the predictive performance of the existing TAVI CPMs, suggesting that CSHA-estimated frailty is limited in augmenting TAVI risk stratification. Arguably, one should regard frailty as a spectrum, rather than a binary phenomenon; however, the sample size of the current study restricted our ability to sub-classify KATZ and CSHA into their individual components. Moreover, the marginal improvement in predictive demonstrated after the inclusion of frailty/disability measures into existing TAVI CPMs, suggests that these measures cannot overcome the need to discover novel risk factors in this patient cohort, or the need to predict endpoints other than mortality (e.g. readmission or quality of life).

Indeed, outcomes after TAVI should consider both mortality and quality of life in the context of the elderly patients that predominantly undergo TAVI. Previous work has shown associations between frailty and quality of life [37] and a contemporary TAVI CPM that was derived to predict poor outcome (defined as mortality and/or reduced quality of life) included measures of functional status and cognitive decline [38]. An external validation of this latter model highlighted a small incremental improvement in performance by adding frailty measures, which were not available at the time of initial development [38,39]. Within the current study, indicators of quality of life were not available, which should be noted as a limitation of the study. Consequently, further studies are needed to investigate the effect of frailty on endpoints such as quality of life or hospital re-admission, which are increasingly used as a measure of futility in TAVI patients.

Nevertheless, identification of futile TAVI cases by using subjective measures of frailty could be problematic in situations of low inter-rater reliability. The current study demonstrated large spatial variability in the proportion of TAVI procedures conducted on ‘frail’ patients, but the subjectivity of CSHA and KATZ means that we were unable to separate genuine spatial differences from systematic variation in how different teams define/record these measures. This provides further indication for the need to refine the

For peer review only

For peer review only - http://bmjopen.bmj.com/site/about/guidelines.xhtml

assessment of frailty in TAVI patients, since the current methods were derived for different purposes. Similarly, we observed an inverse relationship between increasing centre-volume and proportions of poor mobility and KATZ<6 procedures. Decisions around the management of aortic stenosis and the referral for a TAVI are often made following discussions between cardiothoracic surgeons and cardiologists during the heart team assessment. Therefore, the surgical AVR vs TAVI case mix within each individual centre will reflect a complex dynamic driven by the availability of local services and resources, as well as the differences in practice of cardiothoracic surgeons and cardiologists who form the TAVI heart team. While high volume centres may have embraced the treatment of intermediate risk patients with TAVI, lower volume centres may continue to focus on the higher risk patients that are not considered surgical candidates. Given the rapidly changing evidence basis, this is likely to represent a dynamic landscape.

Study Limitations

The strength of this study is that it is a large, contemporary study, which has investigated the impact of frailty on outcomes and mortality prediction post TAVI from a national perspective. However, several limitations need to be considered. Firstly, only three frailty measures were available, with objective frailty tests such as 6-min walk distance, grip strength or gait speed not recorded. Secondly, unmeasured confounders inherent in most observational studies potentially influence conclusions. Thirdly, given that we could only analyse TAVI patients in 2013/14, this retrospective study was underpowered to investigate endpoints with small event rates such as bleeding or myocardial infarction. As the volume of frailty data increases, future studies will be able to investigate such outcomes. Finally, given that this was a retrospective analysis of a national TAVI registry, we could not examine frailty in untreated aortic stenosis patients, and neither could we compare the outcomes with patients treated through surgical aortic valve replacement. Thus, associations between frailty

For peer review only - http://bmjopen.bmj.com/site/about/guidelines.xhtml
For peer review only

and the propensity for conservative treatment could not be explored, with corresponding potential for selection bias. Specifically, the analysis highlights potential measures to predict the expected outcomes after TAVI, but not the expected outcomes without TAVI.

Conclusions

Frailty and disability, as estimated by KATZ, CSHA and poor mobility, was significantly associated with mortality and morbidity after TAVI. The predictive performance and patient-level risk-stratification agreement was significantly improved by updating existing TAVI CPMs to include measures of frailty/ ADL dependency. Hence, physician-estimated frailty/disability measures could aid TAVI risk stratification, until scales that are more objective are routinely collected.

Acknowledgements

We would like to acknowledge all participating centres for collecting the data and the National Institute for Cardiovascular Outcomes Research (NICOR) for provided the UK TAVI registry extract for this study.

Funding Statement

This research was funded by the Medical Research Council, through the Health e-Research Centre, University of Manchester [MR/K006665/1] and the North Staffordshire Heart Committee.

Competing Interests Statement

The authors have no conflict of interest.

For peer review only - http://bmjopen.bmj.com/site/about/guidelines.xhtml

Author Contributions

GPM, MS and MAM made substantial contributions to the concept of the work in addition to performing the analysis. GPM drafted the initial version of the manuscript. GPM, MS, PFL, MAB. MG, JT, SR, UK, IB, and MAM interpreted the results, revised the paper critically for important intellectual content and approved the final version of the paper. All authors agreed to be accountable for all aspects of the work.

For peer review only

For peer review only - http://bmjopen.bmj.com/site/about/guidelines.xhtml

References

1 Leon M, Smith C, Mack M, *et al.* Transcatheter aortic-valve implantation for aortic stenosis in patients who cannot undergo surgery. *N Engl J Med* 2010;**363**:1597–607.

2 Smith C, Leon M, Mack M, *et al.* Transcatheter versus surgical aortic-valve replacement in high-risk patients. *N Engl J Med* 2011;**364**:2187–98.

3 Leon MB, Smith CR, Mack MJ, *et al.* Transcatheter or Surgical Aortic-Valve Replacement in Intermediate-Risk Patients. *N Engl J Med* 2016;**374**:1609–20. doi:10.1056/NEJMoa1514616

4 Vahanian A, Alfieri O, Andreotti F, *et al.* Guidelines on the management of valvular heart disease (version 2012): The Joint Task Force on the Management of Valvular Heart Disease of the European Society of Cardiology (ESC) and the European Association for Cardio-Thoracic Surgery (EACTS). *Eur Heart J* 2012;**33**:2451–96. doi:10.1093/eurheartj/ehs109

5 Iung B, Laouénan C, Himbert D, *et al.* Predictive factors of early mortality after transcatheter aortic valve implantation: individual risk assessment using a simple score. *Heart* 2014;**100**:1016–23. doi:10.1136/heartjnl-2013-305314

6 Capodanno D, Barbanti M, Tamburino C, *et al.* A simple risk tool (the OBSERVANT score) for prediction of 30-day mortality after transcatheter aortic valve replacement. *Am J Cardiol* 2014;**113**:1851–8. doi:10.1016/j.amjcard.2014.03.014

7 Edwards FH, Cohen DJ, O'Brien SM, *et al.* Development and Validation of a Risk Prediction Model for In-Hospital Mortality After Transcatheter Aortic Valve Replacement. *JAMA Cardiol* 2016;**1**:46. doi:10.1001/jamacardio.2015.0326

8 Fried LP, Tangen CM, Walston J, *et al.* Frailty in Older Adults: Evidence for a Phenotype. *Journals Gerontol Ser A Biol Sci Med Sci* 2001;**56**:M146–57.

For peer review only

For peer review only - http://bmjopen.bmj.com/site/about/guidelines.xhtml

doi:10.1093/gerona/56.3.M146

9 Lee DH, Buth KJ, Martin B-J, *et al.* Frail patients are at increased risk for mortality and prolonged institutional care after cardiac surgery. *Circulation* 2010;**121**:973–8. doi:10.1161/CIRCULATIONAHA.108.841437

10 Afilalo J, Mottillo S, Eisenberg MJ, *et al.* Addition of frailty and disability to cardiac surgery risk scores identifies elderly patients at high risk of mortality or major morbidity. *Circ Cardiovasc Qual Outcomes* 2012;**5**:222–8. doi:10.1161/CIRCOUTCOMES.111.963157

11 Puls M, Sobisiak B, Bleckmann A, *et al.* Impact of frailty on short- and long-term morbidity and mortality after transcatheter aortic valve implantation: risk assessment by Katz Index of activities of daily living. *EuroIntervention* 2014;**10**:609–19. doi:10.4244/EIJY14M08_03

12 Rodés-Cabau J, Webb JG, Cheung A, *et al.* Long-term outcomes after transcatheter aortic valve implantation: Insights on prognostic factors and valve durability from the Canadian Multicenter Experience. *J Am Coll Cardiol* 2012;**60**:1864–75. doi:10.1016/j.jacc.2012.08.960

13 Cockburn J, Singh MS, Rafi NHM, *et al.* Poor mobility predicts adverse outcome better than other frailty indices in patients undergoing transcatheter aortic valve implantation. *Catheter Cardiovasc Interv* 2015;**86**:1271–7. doi:10.1002/ccd.25991

14 Afilalo J, Lauck S, Kim DH, *et al.* Frailty in Older Adults Undergoing Aortic Valve Replacement: The FRAILTY-AVR Study. *J Am Coll Cardiol* 2017;**70**:689–700. doi:10.1016/j.jacc.2017.06.024

15 Shimura T, Yamamoto M, Kano S, *et al.* Impact of the Clinical Frailty Scale on Outcomes After Transcatheter Aortic Valve ReplacementClinical Perspective. *Circulation* 2017;**135**:2013–24. doi:10.1161/CIRCULATIONAHA.116.025630

For peer review only - http://bmjopen.bmj.com/site/about/guidelines.xhtml

16 Kappetein AP, Head SJ, Genereux P, *et al.* Updated standardized endpoint definitions for transcatheter aortic valve implantation: the Valve Academic Research Consortium-2 consensus document. *Eur Heart J* 2012;**33**:2403–18. doi:10.1093/eurheartj/ehs255

17 Durand E, Borz B, Godin M, *et al.* Performance analysis of EuroSCORE II compared to the original logistic EuroSCORE and STS scores for predicting 30-day mortality after transcatheter aortic valve replacement. *Am J Cardiol* 2013;**111**:891–7. doi:10.1016/j.amjcard.2012.11.056

18 Martin GP, Sperrin M, Ludman PF, *et al.* Inadequacy of existing clinical prediction models for predicting mortality after transcatheter aortic valve implantation. *Am Heart J* 2017;**184**:97–105. doi:10.1016/j.ahj.2016.10.020

19 Halkin A, Steinvil A, Witberg G, *et al.* Mortality prediction following transcatheter aortic valve replacement: A quantitative comparison of risk scores derived from populations treated with either surgical or percutaneous aortic valve replacement. The Israeli TAVR Registry Risk Model Accuracy A. *Int J Cardiol* 2016;**215**:227–31. doi:10.1016/j.ijcard.2016.04.038

20 Ludman PF. The UK transcatheter aortic valve implantation registry; one of the suite of registries hosted by the National Institute for Cardiovascular Outcomes Research (NICOR). *Heart* 2012;**98**:1787–9. doi:10.1136/heartjnl-2012-302534

21 Rockwood K, Song X, MacKnight C, *et al.* A global clinical measure of fitness and frailty in elderly people. *C Can Med Assoc J* 2005;**173**:489–95. doi:10.1503/cmaj.050051

22 Katz S. Studies of Illness in the Aged. The Index of ADL: A Standardized Measure of Biological and Psychosocial Function. *JAMA* 1963;**185**:914. doi:10.1001/jama.1963.03060120024016

23 Nashef SAM, Roques F, Sharples LD, *et al.* EuroSCORE II. *Eur J Cardio-Thoracic*

For peer review only - http://bmjopen.bmj.com/site/about/guidelines.xhtml

Surg 2012;**41**:734–45. doi:10.1093/ejcts/ezs043

24 Rubin DB. *Multiple Imputation for Nonresponse in Surveys*. John Wiley & Sons 1987.

25 Sterne JAC, White IR, Carlin JB, *et al.* Multiple imputation for missing data in epidemiological and clinical research: potential and pitfalls. *BMJ* 2009;**338**:b2393–b2393. doi:10.1136/bmj.b2393

26 von Hippel PT. Regression with Missing YS: An Improved Strategy For Analyzing Multiply Imputed Data. *Sociol Methodol* 2007;**37**:83–117. doi:10.1111/j.1467-9531.2007.00180.x

27 Steyerberg EW, Borsboom GJJM, van Houwelingen HC, *et al.* Validation and updating of predictive logistic regression models: a study on sample size and shrinkage. *Stat Med* 2004;**23**:2567–86. doi:10.1002/sim.1844

28 Janssen KJM, Moons KGM, Kalkman CJ, *et al.* Updating methods improved the performance of a clinical prediction model in new patients. *J Clin Epidemiol* 2008;**61**:76–86. doi:10.1016/j.jclinepi.2007.04.018

29 Steyerberg EW. *Clinical Prediction Models*. Springer New York 2009.

30 DeLong ER, DeLong DM, Clarke-Pearson DL. Comparing the Areas under Two or More Correlated Receiver Operating Characteristic Curves: A Nonparametric Approach. *Biometrics* 1988;**44**:837. doi:10.2307/2531595

31 R Core Team R. R: A Language and Environment for Statistical Computing. R Found. Stat. Comput. 2017. doi:10.1007/978-3-540-74686-7

32 Wickham H. *ggplot2: elegant graphics for data analysis*. Springer New York 2009.

33 Buuren S Van, Groothuis-Oudshoorn K. mice: Multivariate Imputation by Chained Equations in R. *J Stat Softw* 2011;**45**:1–67.http://www.jstatsoft.org/v45/i03/

34 Alfredsson J, Stebbins A, Brennan JM, *et al.* Gait speed predicts 30-day mortality after

For peer review only - http://bmjopen.bmj.com/site/about/guidelines.xhtml

transcatheter aortic valve replacement: Results from the Society of Thoracic Surgeons/American College of Cardiology Transcatheter Valve Therapy Registry. *Circulation* 2016;**133**:1351–9. doi:10.1161/CIRCULATIONAHA.115.020279

35 Green P, Woglom AE, Genereux P, *et al.* The impact of frailty status on survival after transcatheter aortic valve replacement in older adults with severe aortic stenosis: A single-center experience. *JACC Cardiovasc Interv* 2012;**5**:974–81. doi:10.1016/j.jcin.2012.06.011

36 Nishimura RA, Otto CM, Bonow RO, *et al.* 2014 AHA/ACC Guideline for the Management of Patients With Valvular Heart Disease. *Circulation* 2014;**129**:e521 LP-e643.

37 Green P, Arnold S V., Cohen DJ, *et al.* Relation of Frailty to Outcomes After Transcatheter Aortic Valve Replacement (from the PARTNER Trial). *Am J Cardiol* 2015;**116**:264–9. doi:10.1016/j.amjcard.2015.03.061

38 Arnold S V., Reynolds MR, Lei Y, *et al.* Predictors of poor outcomes after transcatheter aortic valve replacement results from the PARTNER (Placement of Aortic Transcatheter Valve) trial. *Circulation* 2014;**129**:2682–90. doi:10.1161/CIRCULATIONAHA.113.007477

39 Arnold S V., Afilalo J, Spertus JA, *et al.* Prediction of Poor Outcome After Transcatheter Aortic Valve Replacement. *J Am Coll Cardiol* 2016;**68**:1868–77. doi:10.1016/j.jacc.2016.07.762

For peer review only

For peer review only - http://bmjopen.bmj.com/site/about/guidelines.xhtml

Figure Legends

Figure 1: Venn diagram showing the overlap in the different definitions of frailty/disability across CSHA-estimated frailty, KATZ<6 and physician-estimated poor mobility; the sizes of each segment are proportional to the segment sample sizes.

Figure 2: Proportion of patients classified as Non-Frail, Moderately Frail or Severely Frail per total centre volume for the composite frailty score. Definitions of each frailty group are described in Table 1.

Figure 3: Kaplan-Meier plots across CSHA-estimated frailty (top-left), KATZ activities of daily living dependency (top-right), physician-estimated poor mobility (bottom-left), and the composite frailty score (bottom-right). Definitions are described in Table 1.

For peer review only

Tables

Table 1. Frailty grouping definitions by each frailty/disability measure.

Frailty/disability Measure	Scoring System	Frailty definition used in this analysis
CSHA*	A physician-estimated frailty score, based on the following options: 1. Very fit; 2. Well; 3. Well with treated comorbid disease; 4. Apparently vulnerable; 5. Mildly frail with limited dependence for activities of daily living (ADL); 6. Moderately frail requiring help with ADL; 7. Severely frail being completely dependent.	CSHA options of 5, 6, and 7 were used to define CSHA Frail patients; options 1, 2, 3 and 4 were classed as CSHA Non-Frail.
KATZ*	0 to 6-point scale assessing dependency in the following ADL: 1. Bathing; 2. Dressing; 3. Toileting; 4. Transferring; 5. Continence; 6. Feeding.	Any patient with a KATZ Score < 6 points compared with those with KATZ=6.
Poor Mobility	A physician-estimated indication of any severe impairment of mobility that is secondary to musculoskeletal or neurological dysfunction.	Any patient defined as having poor mobility compared with those defined as having normal mobility.
Composite Score	Combination of CSHA, KATZ and poor mobility.	Defined as the following:<br>(i) Non-Frail if a patient is estimated as not frail by CSHA, KATZ and poor mobility;<br>(ii) Moderately Frail if a patient is estimated as frail by at least one (but not all) considered frailty

For peer review only - http://bmjopen.bmj.com/site/about/guidelines.xhtml

		measures;<br>(iii) Severely Frail if a patient is classed as frail across all three of CSHA, KATZ and poor mobility.

**: dichotomising of CSHA and KATZ into two groups was based on the median level observed in the TAVI registry (see Supplementary Figures 1 and 2 for the patient distribution of these scores).*

For peer review only

Table 2. Baseline characteristics as a whole cohort and across CSHA-estimated frailty groups.

**Variable**	**Whole Cohort (n=2624)**	**CSHA-estimated Frail (n=1043)**	**CSHA-estimated Non-Frail (n=1581)**	**p-value**
Age, mean (SD)	81.2 (7.58)	82.0 (7.13)	80.7 (7.82)	<0.001
Female, n (%)	1192 (45.4)	525 (50.3)	667 (42.2)	<0.001
Diabetic, n (%)	641 (24.4)	280 (26.8)	361 (22.8)	0.021
Smoker, n (%)	1316 (50.2)	518 (49.7)	798 (50.5)	0.962
Creatinine, mean (SD)	110.9 (60.5)	116.5 (67.0)	107.2 (55.6)	<0.001
Renal Failure *, n (%)	143 (5.45)	72 (6.90)	71 (4.49)	0.010
Previous MI, n (%)	558 (21.3)	246 (23.6)	312 (19.7)	0.021
Pulmonary Disease, n (%)	791 (30.1)	321 (30.8)	470 (29.7)	0.564
Neurological Disease, n (%)	441 (16.8)	199 (19.1)	242 (15.3)	0.014
Extracardiac Arteriopathy, n (%)	555 (21.2)	268 (25.7)	287 (18.2)	<0.001
Calcification of Ascending Aorta, % (n)	378 (14.4)	116 (11.1)	262 (16.6)	<0.001
Atrial Fibrillation, n (%)	700 (26.7)	296 (28.4)	404 (25.6)	0.138
Previous Cardiac Surgery, n (%)	799 (30.4)	276 (26.5)	523 (33.1)	<0.001
Previous PCI, n (%)	524 (20.0)	201 (19.3)	323 (20.4)	0.501

For peer review only - http://bmjopen.bmj.com/site/about/guidelines.xhtml

Height, mean (SD)	1.64 (0.10)	1.63 (0.10)	1.65 (0.10)	<0.001
Weight, mean (SD)	75.0 (17.1)	73.7 (17.5)	75.8 (16.7)	0.003
CCS Class 4, n (%)	24 (0.91)	12 (1.15)	12 (0.76)	0.412
NYHA ≥ III, n (%)	1985 (75.6)	922 (88.4)	1063 (67.2)	<0.001
Aortic Valve Area, mean (SD)	0.69 (0.23)	0.68 (0.22)	0.69 (0.24)	0.092
Aortic Valve Peak Gradient, mean (SD)	71.2 (26.2)	67.4 (26.2)	73.7 (25.9)	<0.001
LVEF < 50%, n (%)	948 (36.1)	416 (39.9)	532 (33.6)	0.001
One or more diseased vessels, n (%)	1058 (40.3)	439 (42.1)	619 (39.2)	0.190
Left Main Stem Disease, n (%)	103 (3.93)	56 (5.37)	47 (2.97)	0.002
Non-elective Procedure, n (%)	365 (13.9)	199 (19.1)	166 (10.5)	<0.001
Access Site				
Transfemoral, n (%)	2127 (81.1)	840 (80.5)	1287 (81.4)	0.546
Transapical, n (%)	249 (9.49)	91 (8.72)	158 (9.99)	0.302
Subclavian, n (%)	85 (3.24)	34 (3.26)	51 (3.23)	0.999
Other, n (%)	160 (6.10)	78 (7.48)	82 (5.19)	0.021

** Defined as creatinine >200µmol/l or dialysis for renal failure. LVEF: Left Ventricular Ejection Fraction, MI: Myocardial Infarction, NYHA: New York Heart Association, PCI: Percutaneous coronary intervention.*

For peer review only - http://bmjopen.bmj.com/site/about/guidelines.xhtml

Table 3. Baseline characteristics across frailty groups defined with KATZ<6.

Variable	KATZ<6 (n=846)	KATZ=6 (n=1778)	p-value
Age, mean (SD)	81.5 (7.38)	81.1 (7.67)	0.199
Female, n (%)	417 (49.3)	775 (43.6)	0.007
Diabetic, n (%)	217 (25.7)	424 (23.8)	0.347
Smoker, n (%)	434 (51.3)	882 (49.6)	0.252
Creatinine, mean (SD)	113.6 (69.1)	109.6 (56.0)	0.121
Renal Failure *, n (%)	48 (5.67)	95 (5.34)	0.783
Previous MI, n (%)	198 (23.4)	360 (20.2)	0.075
Pulmonary Disease, n (%)	279 (33.0)	512 (28.8)	0.028
Neurological Disease, n (%)	159 (18.8)	282 (15.9)	0.069
Extracardiac Arteriopathy, n (%)	184 (21.7)	371 (20.9)	0.635
Calcification of Ascending Aorta, % (n)	134 (15.8)	244 (13.7)	0.167
Atrial Fibrillation, n (%)	263 (31.1)	437 (24.6)	<0.001
Previous Cardiac Surgery, n (%)	228 (27.0)	571 (32.1)	0.009
Previous PCI, n (%)	165 (19.5)	359 (20.2)	0.725
Height, mean (SD)	1.64 (0.10)	1.65 (0.10)	0.227
Weight, mean (SD)	75.1 (17.0)	74.9 (17.1)	0.843
CCS Class 4, n (%)	10 (1.18)	14 (0.79)	0.439
NYHA ≥ III, n (%)	677 (80.0)	1308 (73.6)	<0.001
Aortic Valve Area, mean (SD)	0.68 (0.23)	0.69 (0.23)	0.536
Aortic Valve Peak Gradient, mean (SD)	70.4 (24.4)	71.6 (27.1)	0.271
LVEF < 50%, n (%)	356 (42.1)	592 (33.3)	<0.001
One or more diseased vessels, n (%)	352 (41.6)	706 (39.7)	0.416
Left Main Stem Disease, n (%)	36 (4.26)	67 (3.77)	0.655
Non-elective Procedure, n (%)	161 (19.0)	204 (11.5)	<0.001
Access Site			
Transfemoral, n (%)	704 (83.2)	1423 (80.0)	0.070
Transapical, n (%)	76 (8.98)	173 (9.73)	0.581
Subclavian, n (%)	22 (2.60)	63 (3.54)	0.244
Other, n (%)	44 (5.20)	116 (6.52)	0.213

** Defined as creatinine >200μmol/l or dialysis for renal failure. LVEF: Left Ventricular Ejection Fraction, MI: Myocardial Infarction, NYHA: New York Heart Association, PCI: Percutaneous coronary intervention.*

For peer review only - http://bmjopen.bmj.com/site/about/guidelines.xhtml

Table 4. Baseline characteristics across those with/without physician-estimated poor mobility.

**Variable**	**Poor Mobility (n=591)**	**Normal Mobility (n=2033)**	**p-value**
Age, mean (SD)	81.4 (7.92)	81.1 (7.47)	0.481
Female, n (%)	328 (55.5)	864 (42.5)	<0.001
Diabetic, n (%)	151 (25.5)	490 (24.1)	0.496
Smoker, n (%)	288 (48.7)	1028 (50.6)	0.677
Creatinine, mean (SD)	115.3 (80.3)	109.6 (53.4)	0.045
Renal Failure *, n (%)	42 (7.11)	101 (4.97)	0.055
Previous MI, n (%)	128 (21.7)	430 (21.2)	0.844
Pulmonary Disease, n (%)	206 (34.9)	585 (28.8)	0.004
Neurological Disease, n (%)	112 (19.0)	329 (16.2)	0.129
Extracardiac Arteriopathy, n (%)	165 (27.9)	390 (19.2)	<0.001
Calcification of Ascending Aorta, % (n)	91 (15.4)	287 (14.1)	0.371
Atrial Fibrillation, n (%)	168 (28.4)	532 (26.2)	0.287
Previous Cardiac Surgery, n (%)	131 (22.2)	668 (32.9)	<0.001
Previous PCI, n (%)	93 (15.7)	431 (21.2)	0.004
Height, mean (SD)	1.63 (0.10)	1.65 (0.10)	<0.001
Weight, mean (SD)	74.8 (17.9)	75.0 (16.8)	0.818
CCS Class 4, n (%)	7 (1.18)	17 (0.84)	0.590
NYHA ≥ III, n (%)	489 (82.7)	1496 (73.6)	<0.001
Aortic Valve Area, mean (SD)	0.67 (0.23)	0.69 (0.23)	0.110
Aortic Valve Peak Gradient, mean (SD)	69.3 (25.3)	71.8 (26.5)	0.050
LVEF < 50%, n (%)	224 (37.9)	724 (35.6)	0.308
One or more diseased vessels, n (%)	231 (39.1)	827 (40.7)	0.551
Left Main Stem Disease, n (%)	18 (3.05)	85 (4.18)	0.282
Non-elective Procedure, n (%)	120 (20.3)	245 (12.1)	<0.001
Access Site			
Transfemoral, n (%)	454 (76.8)	1673 (82.3)	0.004
Transapical, n (%)	80 (13.5)	169 (8.31)	<0.001
Subclavian, n (%)	23 (3.89)	62 (3.05)	0.374
Other, n (%)	33 (5.58)	127 (6.25)	0.623

** Defined as creatinine >200μmol/l or dialysis for renal failure. LVEF: Left Ventricular Ejection Fraction, MI: Myocardial Infarction, NYHA: New York Heart Association, PCI: Percutaneous coronary intervention.*

For peer review only - http://bmjopen.bmj.com/site/about/guidelines.xhtml

Table 5. Short-term outcomes across CSHA-estimated frailty, KATZ ADL dependency and physician-estimated poor mobility groups.

**Outcome**	**CSHA-estimated frail (n=1043)**	**CSHA-estimated non-frail (n=1581)**	**Univariable OR (95% CI)***	**Multivariable OR (95% CI)***
30-day mortality	57/1043 (5.47%)	51/1581 (3.23%)	**1.73 (1.18, 2.55)**	1.46 (0.96, 2.23)
Early Safety	187/1014 (18.44%)	190/1540 (12.34%)	**1.61 (1.29, 2.00)**	**1.45 (1.14, 1.84)**
**Outcome**	**KATZ<6 (n=846)**	**KATZ=6 (n=1778)**	**Univariable OR (95% CI)***	**Multivariable OR (95% CI)***
30-day mortality	58/846 (6.86%)	50/1778 (2.81%)	**2.54 (1.73, 3.75)**	**2.10 (1.39, 3.15)**
Early Safety	150/827 (18.14%)	227/1727 (13.14%)	**1.46 (1.17, 1.83)**	**1.28 (1.01, 1.63)**
**Outcome**	**Poor mobility (n=591)**	**Normal mobility (n=2033)**	**Univariable OR (95% CI)***	**Multivariable OR (95% CI)***
30-day mortality	46/591 (7.78%)	62/2033 (3.05%)	**2.68 (1.81, 3.98)**	**2.15 (1.41, 3.28)**
Early Safety	116/571 (20.32%)	261/1983 (13.16%)	**1.68 (1.32, 2.14)**	**1.45 (1.12, 1.88)**

*: *Bold items indicate significant results. CI: Confidence Interval, OR: Odds Ratio*

For peer review only - http://bmjopen.bmj.com/site/about/guidelines.xhtml

Table 6. Time dependent Cox proportional hazards models per frailty indicator.

**Frailty-by-time interaction**	**Univariable HR (95% CI)***	**Multivariable HR (95% CI)***
CSHA-estimated Frail		
0-180 days	**1.56 (1.22, 1.98)**	**1.29 (1.00, 1.66)**
180-365 days	**1.63 (1.12, 2.38)**	1.37 (0.94, 2.01)
>365 days	**1.85 (1.32, 2.60)**	**1.61 (1.14, 2.29)**
KATZ<6		
0-180 days	**1.71 (1.34, 2.18)**	**1.55 (1.21, 1.99)**
180-365 days	**1.86 (1.28, 2.71)**	**1.74 (1.19, 2.55)**
>365 days	1.25 (0.88, 1.78)	1.23 (0.86, 1.75)
Physician-estimated poor Mobility		
0-180 days	**1.74 (1.34, 2.25)**	**1.50 (1.15, 1.96)**
180-365 days	**2.06 (1.39, 3.06)**	**1.84 (1.24, 2.75)**
>365 days	**1.50 (1.03, 2.20)**	1.36 (0.92, 2.00)

**: Bold items indicate significant results.HR: hazard ratio.*

For peer review only

For peer review only - http://bmjopen.bmj.com/site/about/guidelines.xhtml

Table 7. Discrimination of each TAVI CPM at predicting 30-day mortality before and after the addition of each frailty measure.

Model	AUC (95% CI)	p-value*
**FRANCE-2**		
Original	0.62 (0.57, 0.68)	N/A
Updated with CSHA only	0.64 (0.58, 0.69)	0.412
Updated with KATZ only	0.67 (0.61, 0.72)	0.047
Updated with Poor Mobility only	0.67 (0.62, 0.72)	0.058
Updated with stepwise selection †	0.68 (0.63, 0.73)	0.025
**OBSERVANT**		
Original	0.56 (0.50, 0.62)	N/A
Updated with CSHA only	0.59 (0.53, 0.65)	0.129
Updated with KATZ only	0.62 (0.56, 0.68)	0.007
Updated with Poor Mobility only	0.63 (0.57, 0.69)	0.006
Updated with stepwise selection †	0.64 (0.58, 0.71)	0.002
**ACC**		
Original	0.63 (0.58, 0.68)	N/A
Updated with CSHA only	0.64 (0.59, 0.69)	0.605
Updated with KATZ only	0.67 (0.61, 0.72)	0.069
Updated with Poor Mobility only	0.68 (0.63, 0.73)	0.030
Updated with stepwise selection †	0.68 (0.63, 0.74)	0.036

** the DeLong p-value compares the AUC of predicting 30-day mortality using the original TAVI CPM with that using each corresponding updated version. †: forward selection across the three frailty measures resulted in KATZ<6 and physician-estimated poor mobility being added into each existing TAVI CPM.*

For peer review only - http://bmjopen.bmj.com/site/about/guidelines.xhtml

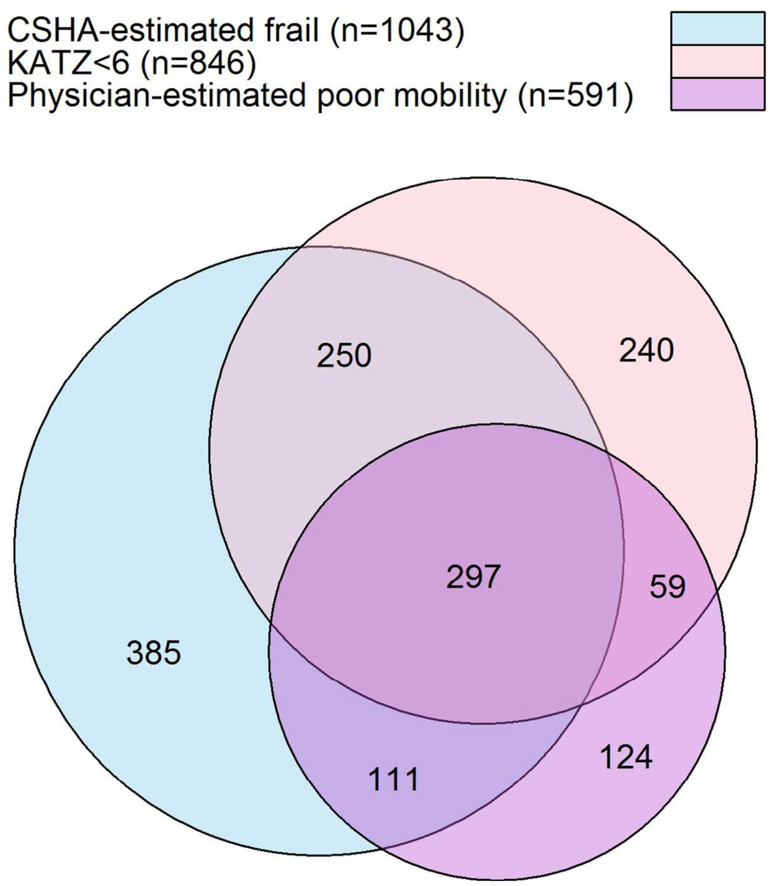

Venn diagram showing the overlap in the different definitions of frailty/disability across CSHA-estimated frailty, KATZ<6 and physician-estimated poor mobility; the sizes of each segment are proportional to the segment sample sizes.

127x127mm (300 x 300 DPI)

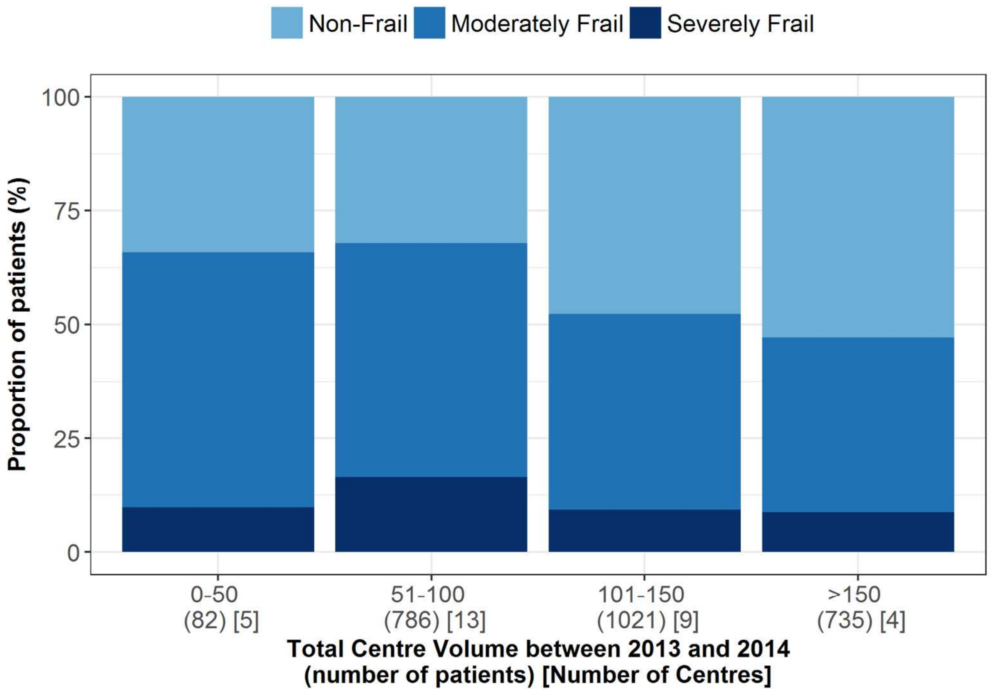

Proportion of patients classified as Non-Frail, Moderately Frail or Severely Frail per total centre volume for the composite frailty score. Definitions of each frailty group are described in Table 1.

177x127mm (300 x 300 DPI)

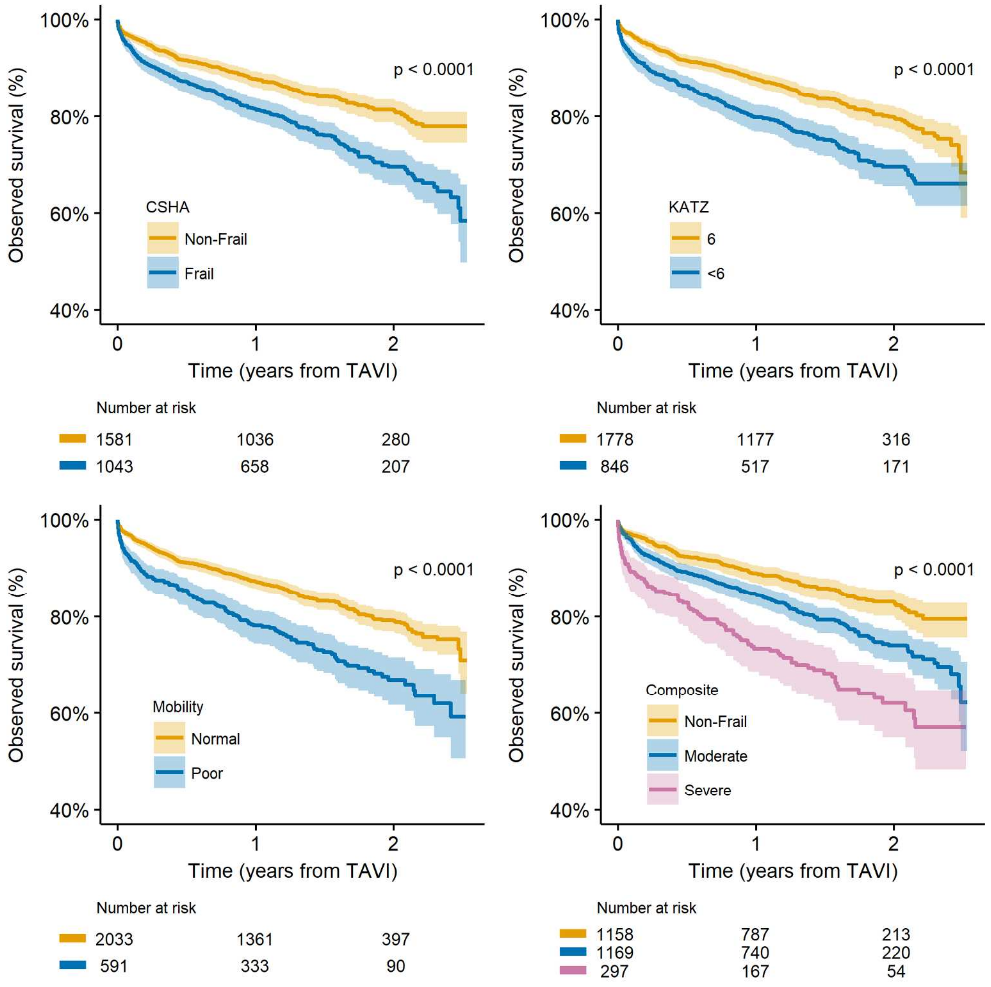

Kaplan-Meier plots across CSHA-estimated frailty (top-left), KATZ activities of daily living dependency (top-right), physician-estimated poor mobility (bottom-left), and the composite frailty score (bottom-right). Definitions are described in Table 1.

177x177mm (300 x 300 DPI)

Supplementary Material

Frailty measures are key to better prediction of mortality and morbidity following transcatheter aortic valve implantation

Glen P. Martin; Matthew Sperrin; Peter F. Ludman; Mark A. de Belder; Mark Gunning; John Townend; Simon Redwood; Umesh Kadam; Iain Buchan; Mamas A. Mamas

Supplementary Methods

Mathematical Details of Model Updating Techniques

Let P denote the number of predictors that are included in an existing TAVI CPM. Then the jth existing TAVI CPM provides a linear predictor (LP) on the logit scale of the form

$$\mathrm{LP}_{i,j} = \beta_{0,j} + \sum_{p=1}^{P} \beta_{p,j} x_{i,p}$$

for patient i, covariates $x_{i,p}$ for $p \in [1, P]$, and associated coefficients $\beta_{p,j}$.

Then model updating methods, as previously described (1–4), have a hierarchical structure to alter an existing CPM to a new population. This study considered the addition of new variables that were not included in the original TAVI CPMs (i.e. frailty). Specifically, model updating of the jth existing TAVI CPM fits a logistic regression model in the UK TAVI registry with the jth existing LP and a frailty indicator as the only covariates, by modelling

$$\log\left(\frac{\pi_i}{1-\pi_i}\right) = \hat{\alpha}_0 + \hat{\alpha}_1 \mathrm{LP}_{i,j} + \delta_{\text{Frailty}} x_{i,\text{Frailty}} \quad (1)$$

where π_i denotes the predicted event probability for patient i in the UK TAVI registry, $x_{i,\text{Frailty}}$ is either KATZ, CSHA or poor mobility indication, and δ_{Frailty} is the corresponding coefficient estimate. The parameters $\hat{\alpha}_0$ and $\hat{\alpha}_1$ are the calibration intercept and slope, respectively.

This process was repeated for all three of the frailty measures individually and across all of the considered TAVI CPMs. Additionally, to explore the incremental benefit of adding the frailty measures into the TAVI CPMs, we iteratively added each of the frailty measures into Equation (1), under a likelihood ratio test selection criterion until all those frailty measures that significantly improved model fit were selected.

All models were fit in each multiple imputed dataset separately, with the coefficient estimates then pooled according to Rubin's rules. Predictions were then made for each patient in the UK registry, using the pooled coefficient estimates. Subsequent performance estimates (e.g. area under the ROC curve) were made in each imputed dataset, which were then pooled using Rubin's rules.

For peer review only

For peer review only

Supplementary Figures

Supplementary Figure 1: Distribution of the CSHA frailty score (left panel) and the crude 30-day mortality rate (right panel)

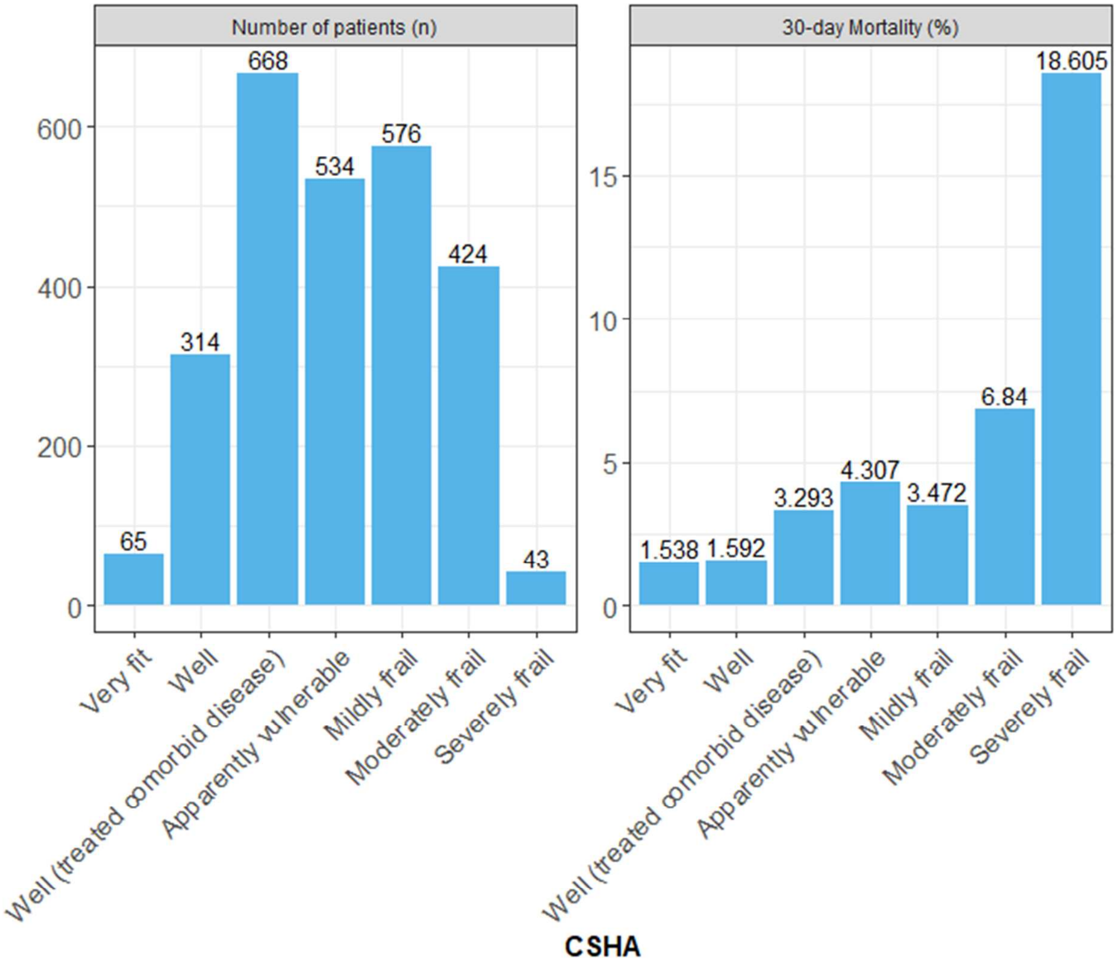

Supplementary Figure 2: Distribution of the KATZ frailty score (left panel) and the crude 30-day mortality rate (right panel)

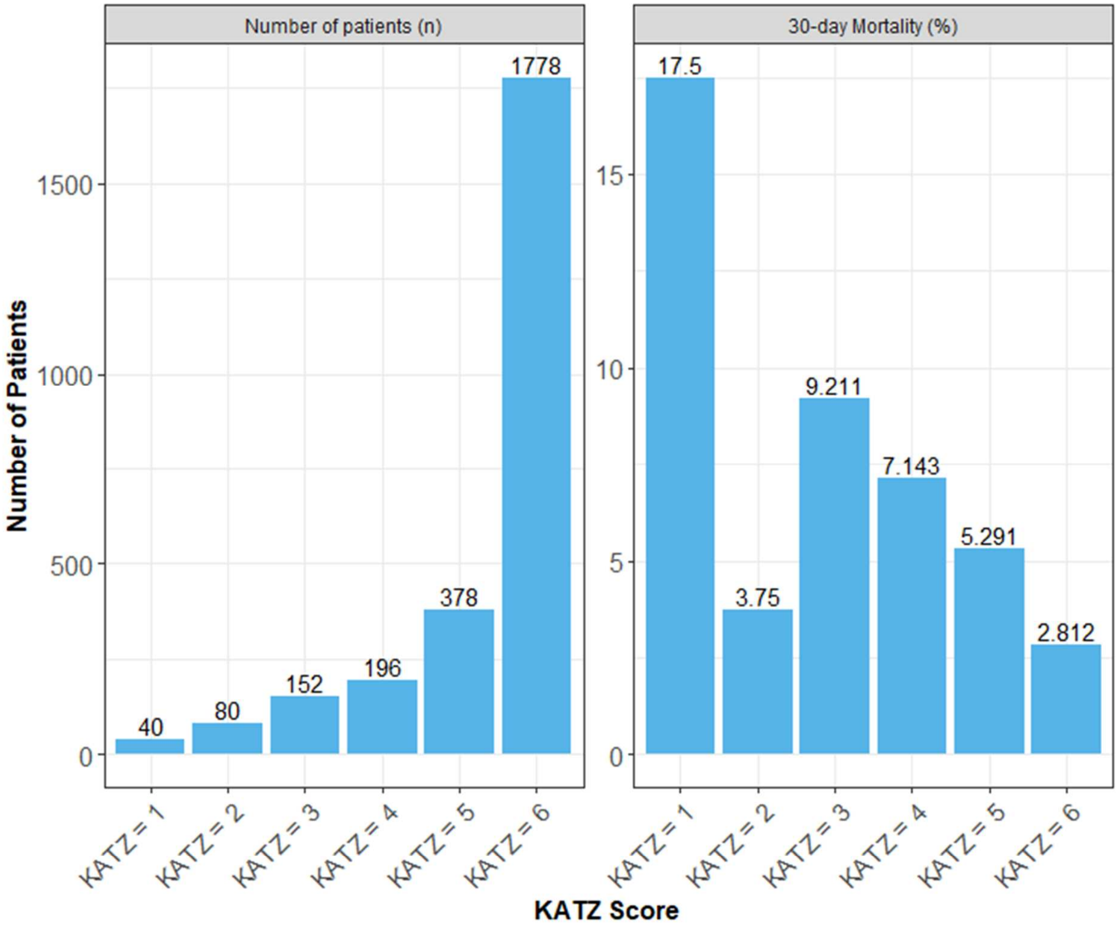

Supplementary Figure 3: Proportion of frail patients across the three frailty measures per total centre volume.

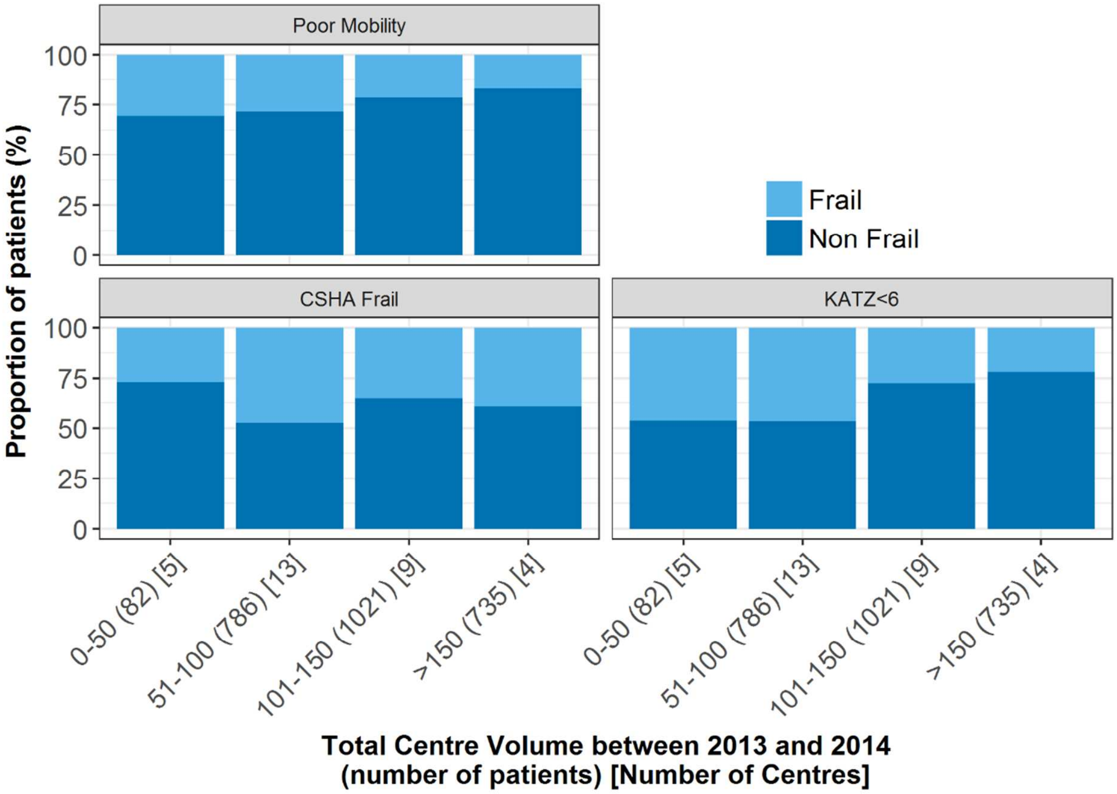

Supplementary Figure 4: Proportion of patients defined as having CSHA-estimated frailty, KATZ activities of daily living dependency, or physician-estimated poor mobility per NHS England region and Wales. The sample size of each region is also depicted.

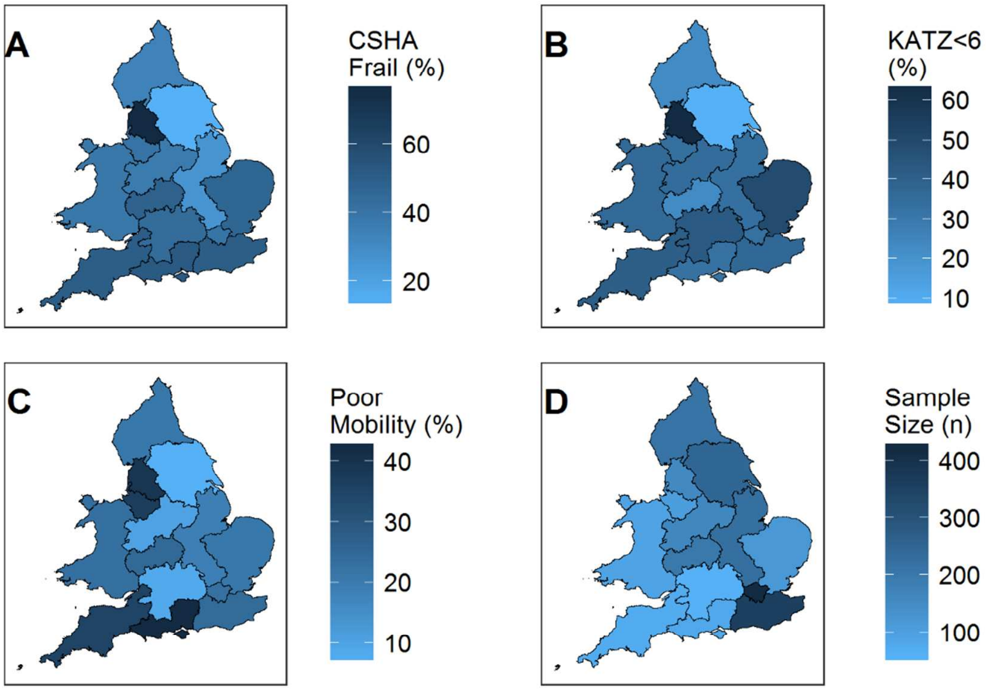

Supplementary Tables

Supplementary Table 1. Variables that were included in the original versions of each of the previously published TAVI clinical prediction models.

**FRANCE-2 Model** (5)	**OBSERVANT Model** (6)	**ACC Model** (7)
Age	Glomerular filtration rate <45 mL/min	Age per 5-year increments
BMI	Critical preoperative state	Glomerular filtration rate per 5-U increments
Respiratory Insufficiency	Pulmonary hypertension	Dialysis vs no dialysis
Acute pulmonary oedema	Diabetes	NYHA class IV
NYHA Class IV	NYHA Class IV	Severe chronic lung disease
Dialysis	Prior BAV	Nonfemoral access site
Pulmonary hypertension	LVEF<40%	Acuity category
Critical preoperative state		
Transapical Access		
Other Access		

For peer review only

Supplementary Table 2. Baseline characteristics across the patients included and excluded in the analysis.

Variable	Included (n=2624)	Excluded (n=449)	p-value
Age, mean (SD)	81.2 (7.58)	80.6 (7.78)	0.127
Female, n (%)	1192 (45.4)	201 (44.8)	0.807
Diabetic, n (%)	641 (24.4)	97 (21.6)	0.573
Smoker, n (%)	1316 (50.2)	193 (43.0)	0.227
Creatinine, mean (SD)	110.9 (60.5)	111.0 (61.2)	0.962
Renal Failure *, n (%)	143 (5.45)	28 (6.24)	0.270
Previous MI, n (%)	558 (21.3)	96 (21.4)	0.506
Pulmonary Disease, n (%)	791 (30.1)	110 (24.5)	0.115
Neurological Disease, n (%)	441 (16.8)	69 (15.4)	0.932
Extracardiac Arteriopathy, n (%)	555 (21.2)	77 (17.1)	0.227
Calcification of Ascending Aorta, % (n)	378 (14.4)	39 (8.69)	0.005
Atrial Fibrillation, n (%)	700 (26.7)	114 (25.4)	0.837
Previous Cardiac Surgery, n (%)	799 (30.4)	109 (24.3)	0.062
Previous PCI, n (%)	524 (20.0)	86 (19.2)	0.903
Height, mean (SD)	1.64 (0.10)	1.66 (0.10)	0.020
Weight, mean (SD)	75.0 (17.1)	76.2 (17.4)	0.209
CCS Class 4, n (%)	24 (0.91)	7 (1.56)	0.243
NYHA ≥ III, n (%)	1985 (75.6)	347 (77.3)	0.001
Aortic Valve Area, mean (SD)	0.69 (0.23)	0.68 (0.23)	0.468
Aortic Valve Peak Gradient, mean (SD)	71.2 (26.2)	72.9 (24.4)	0.243
LVEF < 50%, n (%)	948 (36.1)	161 (35.9)	0.474
One or more diseased vessels, n (%)	1058 (40.3)	151 (33.6)	0.064
Left Main Stem Disease, n (%)	103 (3.93)	18 (4.01)	0.866
Non-elective Procedure, n (%)	365 (13.9)	62 (13.8)	0.999
Access Site			
Transfemoral, n (%)	2127 (81.1)	334 (74.4)	0.005
Transapical, n (%)	249 (9.49)	75 (16.7)	<0.001
Subclavian, n (%)	85 (3.24)	5 (1.11)	0.022
Other, n (%)	160 (6.10)	30 (6.68)	0.674

** Defined as creatinine >200μmol/l or dialysis for renal failure. LVEF: Left Ventricular Ejection Fraction, MI: Myocardial Infarction, NYHA: New York Heart Association, PCI: Percutaneous coronary intervention.*

For peer review only

Supplementary Table 3. Multivariable adjusted short-term outcomes for the sensitivity analysis of the composite frailty indicator. Bold items indicate significant results.

**Composite Frailty**	**Moderately frail vs. non-frail multivariable OR (95% CI) ***	**Severely frail vs. non-frail multivariable OR (95% CI) ***
30-day mortality	1.19 (0.74, 1.92)	**2.99 (1.71, 5.23)**
Early Safety	1.18 (0.91, 1.52)	**1.88 (1.32, 2.68)**

**: Severely Frail (N=297) corresponds to a patient defined as frail across all three of CSHA, KATZ and poor mobility; Moderately Frail (N=1169) corresponds to a patient that is defined frail by at least one (but not all) of CSHA, KATZ and poor mobility; Non-frail (N=1158) means a patient was defined as not frail by all of CSHA, KATZ and poor mobility.*

CI: Confidence Interval, MVC: Major Vascular Complication, OR: Odds Ratio, PPM: Permanent Pacemaker Implantation

For peer review only

Supplementary References

1. Janssen KJM, Moons KGM, Kalkman CJ, Grobbee DE, Vergouwe Y. Updating methods improved the performance of a clinical prediction model in new patients. J Clin Epidemiol. 2008;61(1):76–86.
2. Steyerberg EW, Borsboom GJJM, van Houwelingen HC, Eijkemans MJC, Habbema JDF. Validation and updating of predictive logistic regression models: a study on sample size and shrinkage. Stat Med. 2004;23(16):2567–86.
3. Moons KGM, Kengne AP, Grobbee DE, Royston P, Vergouwe Y, Altman DG, et al. Risk prediction models: II. External validation, model updating, and impact assessment. Heart. 2012;98(9):691–8.
4. Su T-L, Jaki T, Hickey GL, Buchan I, Sperrin M. A review of statistical updating methods for clinical prediction models. Stat Methods Med Res. 2016; DOI: 10.1177/0962280215626466
5. Iung B, Laouénan C, Himbert D, Eltchaninoff H, Chevreul K, Donzeau-Gouge P, et al. Predictive factors of early mortality after transcatheter aortic valve implantation: individual risk assessment using a simple score. Heart. 2014;100(13):1016–23.
6. Capodanno D, Barbanti M, Tamburino C, D'Errigo P, Ranucci M, Santoro G, et al. A simple risk tool (the OBSERVANT score) for prediction of 30-day mortality after transcatheter aortic valve replacement. Am J Cardiol. 2014;113(11):1851–8.
7. Edwards FH, Cohen DJ, O'Brien SM, Peterson ED, Mack MJ, Shahian DM, et al. Development and Validation of a Risk Prediction Model for In-Hospital Mortality After Transcatheter Aortic Valve Replacement. JAMA Cardiol. 2016;1(1):46.

For peer review only

STROBE 2007 (v4) checklist of items to be included in reports of observational studies in epidemiology*
Checklist for cohort, case-control, and cross-sectional studies (combined)

Section/Topic	Item #	Recommendation	Reported on page #
**Title and abstract**	1	(*a*) Indicate the study's design with a commonly used term in the title or the abstract	Title page
		(*b*) Provide in the abstract an informative and balanced summary of what was done and what was found	2
**Introduction**			
Background/rationale	2	Explain the scientific background and rationale for the investigation being reported	5
Objectives	3	State specific objectives, including any pre-specified hypotheses	5
**Methods**			
Study design	4	Present key elements of study design early in the paper	6
Setting	5	Describe the setting, locations, and relevant dates, including periods of recruitment, exposure, follow-up, and data collection	6, 7 and 8
Participants	6	(*a*) *Cohort study*—Give the eligibility criteria, and the sources and methods of selection of participants. Describe methods of follow-up<br>*Case-control study*—Give the eligibility criteria, and the sources and methods of case ascertainment and control selection. Give the rationale for the choice of cases and controls<br>*Cross-sectional study*—Give the eligibility criteria, and the sources and methods of selection of participants	6
		(*b*) *Cohort study*—For matched studies, give matching criteria and number of exposed and unexposed<br>*Case-control study*—For matched studies, give matching criteria and the number of controls per case	N/A
Variables	7	Clearly define all outcomes, exposures, predictors, potential confounders, and effect modifiers. Give diagnostic criteria, if applicable	6 and 7
Data sources/ measurement	8*	For each variable of interest, give sources of data and details of methods of assessment (measurement). Describe comparability of assessment methods if there is more than one group	*6*
Bias	9	Describe any efforts to address potential sources of bias	7 and 8
Study size	10	Explain how the study size was arrived at	N/A – registry data
Quantitative variables	11	Explain how quantitative variables were handled in the analyses. If applicable, describe which groupings were chosen and why	7 and 8
Statistical methods	12	(*a*) Describe all statistical methods, including those used to control for confounding	7 and 8
		(*b*) Describe any methods used to examine subgroups and interactions	7 and 8
		(*c*) Explain how missing data were addressed	7
		(*d*) *Cohort study*—If applicable, explain how loss to follow-up was addressed<br>*Case-control study*—If applicable, explain how matching of cases and controls was addressed	N/A

For peer review only

		Cross-sectional study—If applicable, describe analytical methods taking account of sampling strategy	
		(*e*) Describe any sensitivity analyses	N/A
**Results**			
Participants	13*	(a) Report numbers of individuals at each stage of study—eg numbers potentially eligible, examined for eligibility, confirmed eligible, included in the study, completing follow-up, and analysed	9
		(b) Give reasons for non-participation at each stage	9
		(c) Consider use of a flow diagram	N/A
Descriptive data	14*	(a) Give characteristics of study participants (eg demographic, clinical, social) and information on exposures and potential confounders	Table 2
		(b) Indicate number of participants with missing data for each variable of interest	9
		(c) *Cohort study*—Summarise follow-up time (eg, average and total amount)	N/A
Outcome data	15*	*Cohort study*—Report numbers of outcome events or summary measures over time	Table 5 and throughout results section
		Case-control study—Report numbers in each exposure category, or summary measures of exposure	N/A
		Cross-sectional study—Report numbers of outcome events or summary measures	N/a
Main results	16	(*a*) Give unadjusted estimates and, if applicable, confounder-adjusted estimates and their precision (eg, 95% confidence interval). Make clear which confounders were adjusted for and why they were included	Table 5, Table 6 and page 11
		(*b*) Report category boundaries when continuous variables were categorized	N/A
		(*c*) If relevant, consider translating estimates of relative risk into absolute risk for a meaningful time period	N/A
Other analyses	17	Report other analyses done—eg analyses of subgroups and interactions, and sensitivity analyses	Table 7 and throughout results section
**Discussion**			
Key results	18	Summarise key results with reference to study objectives	13
Limitations	19	Discuss limitations of the study, taking into account sources of potential bias or imprecision. Discuss both direction and magnitude of any potential bias	16 and 17
Interpretation	20	Give a cautious overall interpretation of results considering objectives, limitations, multiplicity of analyses, results from similar studies, and other relevant evidence	14, 15
Generalisability	21	Discuss the generalisability (external validity) of the study results	15
**Other information**			
Funding	22	Give the source of funding and the role of the funders for the present study and, if applicable, for the original study on which the present article is based	17

For peer review only

*Give information separately for cases and controls in case-control studies and, if applicable, for exposed and unexposed groups in cohort and cross-sectional studies.
Note: An Explanation and Elaboration article discusses each checklist item and gives methodological background and published examples of transparent reporting. The STROBE checklist is best used in conjunction with this article (freely available on the Web sites of PLoS Medicine at http://www.plosmedicine.org/, Annals of Internal Medicine at http://www.annals.org/, and Epidemiology at http://www.epidem.com/). Information on the STROBE Initiative is available at www.strobe-statement.org.

For peer review only

Do frailty measures improve prediction of mortality and morbidity following transcatheter aortic valve implantation? An analysis of the UK TAVI registry

Journal:	*BMJ Open*
Manuscript ID	bmjopen-2018-022543.R1
Article Type:	Research
Date Submitted by the Author:	09-Mar-2018

Complete List of Authors:	Martin, Glen; University of Manchester Institute of Population Health, Farr Institute<br>Sperrin, Matthew; University of Manchester Institute of Population Health, Farr Institute<br>Ludman, Peter; University Hospitals Birmingham NHS Foundation Trust, Cardiology<br>deBelder, Mark; South Tees NHS Foundation Trust, Cardiology<br>Gunning, Mark ; Keele University, Keele Cardiovascular Research Group<br>Townend, Jonathan; University Hospitals Birmingham NHS Foundation Trust, Cardiology<br>Redwood, Simon R; St Thomass Hospital<br>Kadam, Umesh; Keele University, Keele Cardiovascular Research Group<br>Buchan, Iain; Microsoft Research<br>Mamas, Mamas; Keele University, Keele Cardiovascular Research Group

**Primary Subject Heading**:	Cardiovascular medicine
Secondary Subject Heading:	Health informatics
Keywords:	Frailty, Transcatheter Aortic Valve Implantation, Mortality, Risk Stratification, Clinical Prediction Models

Clinical Investigation

Do frailty measures improve prediction of mortality and morbidity following transcatheter aortic valve implantation? An analysis of the UK TAVI registry

Short title: Frailty and outcomes following TAVI

Glen P. Martin, PhD [1]; Matthew Sperrin, PhD [1]; Peter F. Ludman, MA, MD, FRCP, FESC [2]; Mark A. de Belder, MA, MD, FRCP [3]; Mark Gunning, MD, FRCP [4]; John Townend, MD [2]; Simon Redwood, MBBS, MD [5]; Umesh Kadam, MD [4]; Iain Buchan, MD, FFPH [1,6]; Mamas A. Mamas, MA, DPhil, FRCP [1,4]

1: Farr Institute, Faculty of Biology, Medicine and Health, University of Manchester, Manchester Academic Health Science Centre, Manchester, United Kingdom

2: Queen Elizabeth Hospital, Birmingham, United Kingdom

3: The James Cook University Hospital, Middlesbrough, United Kingdom

4: Keele Cardiovascular Research Group, Institute of Applied Clinical Science and Centre for Prognosis Research Group, Institute of Primary Care and Health Sciences, Keele University, and Academic Department of Cardiology, Royal Stoke Hospital, Stoke-on-Trent, UK

5: Guys & St Thomas' NHS Foundation Trust, London, UK

6: Microsoft Research, Cambridge, UK

Manuscript Word Count: 3148

Corresponding author:

Dr Glen Philip Martin

Lecturer in Health Data Science

Farr Institute, Faculty of Biology, Medicine and Health, University of Manchester, Manchester, M13 9GB, United Kingdom

Email: glen.martin@manchester.ac.uk

For peer review only - http://bmjopen.bmj.com/site/about/guidelines.xhtml

Structured Abstract

Objectives

Previous studies indicate frailty to be associated with poor outcomes following transcatheter aortic valve implantation (TAVI), but there is limited evidence from multicentre registries. The aim was to investigate the independent association of frailty with TAVI outcomes, and the prognostic utility of adding frailty into existing clinical prediction models (CPMs).

Design

The UK TAVI registry incorporated three frailty measures since 2013: CSHA, KATZ and poor mobility. We investigated the associations between these frailty measures with short- and long-term outcomes, using logistic regression to estimate multivariable adjusted odds ratios (ORs), and Cox proportional hazards models to explore long-term survival. We compared the predictive performance of existing TAVI CPMs before and after updating them to include each frailty measure.

Setting

All patients who underwent a TAVI procedure in England or Wales between 2013 and 2014.

Participants

2624 TAVI procedures were analysed in this study.

Primary and Secondary Outcomes

The primary endpoints in this study were 30-day mortality and long-term survival. The Valve Academic Research Consortium (VARC)-2 composite early safety endpoint was considered as a secondary outcome.

For peer review only - http://bmjopen.bmj.com/site/about/guidelines.xhtml

Results

KATZ<6 (OR 2.10, 95% CI: 1.39, 3.15) and poor mobility (OR 2.15, 95% CI: 1.41, 3.28) predicted 30-day mortality after multivariable adjustment. All frailty measures were associated with increased odds of the Valve Academic Research Consortium (VARC)-2 composite early safety endpoint. We observed a significant increase in the area under the ROC curves by approximately 5% after adding KATZ<6 or poor mobility. Risk stratification agreement was significantly improved by the addition of each frailty measure, with an increase in intra-class correlation coefficient of between 0.15 and 0.31.

Conclusion

Frailty was associated with worse outcomes following TAVI, and incorporating frailty metrics significantly improved the predictive performance of existing CPMs. Physician-estimated frailty measures could aid TAVI risk stratification, until more objective scales are routinely collected.

Keywords

Frailty – Transcatheter Aortic Valve Implantation – Mortality – Risk Stratification – Clinical Prediction Models

For peer review only

For peer review only - http://bmjopen.bmj.com/site/about/guidelines.xhtml

Article Summary

Strengths and Limitations of this Study

- An analysis of the association between three frailty measures and clinical outcomes following transcatheter aortic valve implantation (TAVI).
- The study used data from the UK TAVI registry, including all consecutive patients who underwent TAVI in UK between 2013 and 2014.
- A large, contemporary study, which investigated the impact of frailty on outcomes and mortality prediction post TAVI from a national perspective.
- Only three subjective frailty measures were available, with objective frailty tests not recorded.
- This retrospective analysis could not compare outcomes across frailty groups in untreated aortic stenosis patients, or those undergoing surgical replacement.

For peer review only

For peer review only - http://bmjopen.bmj.com/site/about/guidelines.xhtml

Introduction

Transcatheter aortic valve implantation (TAVI) has emerged as an effective treatment strategy for patients with aortic stenosis who are intermediate-to-high operative risk [1–3]. To this end, surgical CPMs, such as the EuroSCORE or the STS model, have previously been used to estimate risk, but TAVI specific CPMs are emerging from national registries: examples include the FRANCE-2 model [4], the OBSERVANT model [5] and the American College of Cardiology Transcatheter Valve Therapy (ACC) model [6]. However, these models do not usually incorporate measures of frailty and disability into geriatric pre-screening risk assessment.

Frailty is an emerging concept in clinical science and is defined as an age-related decline in the resilience to stressors caused by deterioration in multiple physiological systems [7]. Consequently, there is a growing evidence base of associations between frailty and poor outcomes after TAVI [8–13]. Indeed, patient's frailty, dependencies in activities of daily living (ADL) and cognitive function are increasingly being considered in the decision-making process for TAVI eligibility [14]. However, measures of frailty are rarely incorporated into many of the currently available CPMs, which has potentially contributed to the moderate predictive performance of such models outside their development datasets [15–17]. The recent recording of measures relating to frailty/disability in national cohorts presents novel opportunities to incorporate them into future iterations of existing TAVI CPMs.

Therefore, the aim was to investigate the effect of frailty and disability on mortality and morbidity in a national TAVI cohort and to examine the prognostic utility of adding frailty/disability into existing TAVI CPMs.

For peer review only - http://bmjopen.bmj.com/site/about/guidelines.xhtml

Methods

UK TAVI Registry

The UK TAVI registry prospectively collects 95 variables (including patient demographics, risk factors for intervention and within-hospital adverse outcomes), for every TAVI procedure conducted in the UK [18]. This analysis used data from patients in England and Wales, for whom all-cause mortality was linked from the Office for National Statistics. The study period for this analysis was January 2013 to December 2014, corresponding to the time in which the UK registry recorded three variables that related to frailty/disability. Specifically, the registry recorded the Canadian Study of Health and Aging (CSHA)-estimated frailty scale [19], KATZ ADL dependency [20], and a physician-estimated poor mobility (as defined in the EuroSCORE II model [21]). Frailty groups were defined separately across the three measures with the full definitions of each group given in **Table 1**. Dichotomising CSHA and KATZ scores into two categories was based on the empirical median value (CSHA="apparently vulnerable" and KATZ=6), and on the original publication of CSHA [19]. Additionally, for this analysis, we defined a composite score to incorporate information from all three of the measures, with the definition given in **Table 1**.

Study Endpoints

The primary endpoints in this study were 30-day mortality and long-term survival. As a secondary endpoint, we investigated the Valve Academic Research Consortium (VARC)-2 composite early safety endpoint, which is defined as a failure in any of the following outcomes by 30-days: all-cause mortality, stroke, life-threatening bleeding, stage 2/3 acute kidney injury, coronary artery obstruction requiring intervention, major vascular complications, or valve-related dysfunction requiring a repeat procedure [14].

For peer review only - http://bmjopen.bmj.com/site/about/guidelines.xhtml

Statistical Analysis

Every variable with missing data was imputed using multiple imputation that generated ten imputed datasets [22]. The imputation model for each variable included the majority of other variables in the UK TAVI registry and all considered endpoints [23]. Imputed frailty measures and outcomes were not used, and instead were returned to the original (missing) values [24]. Subsequently, patients missing data on CSHA, KATZ, poor mobility, and/or missing life status were removed from the analysis; patients with missing VARC-2 composite early safety were only removed from the analysis of that outcome. All analyses were undertaken in each imputed dataset separately, with the results pooled according to Rubin's rules [22].

For exploratory analysis, we obtained spatial maps of England at a National Health Service (NHS) regional level (a high-level geographical structure partitioning England into 13 regions) and of Wales, from the Office for National Statistics. By linking the clinical commission group of each patient to the corresponding NHS region, we calculated the proportion of frail patients in the study population across England and Wales.

Odds ratios (ORs) for binary endpoints were estimated using logistic regression, with both unadjusted and adjusted ORs reported. Multivariable models adjusted for the following variables: total centre volume, age, sex, diabetes status, smoking status, creatinine, renal failure, previous myocardial infarction, pulmonary disease, neurological disease, extra-cardiac arteriopathy, calcification of ascending aorta, atrial fibrillation/flutter, previous cardiac surgery, previous percutaneous coronary intervention (PCI), height, weight, critical pre-operative status, Canadian Cardiovascular Society grading class 4, New York Heart Association (NYHA) class ≥III, aortic valve area and peak gradient, Left Ventricular Ejection Fraction (LVEF) <50%, one or more diseases vessels, left main stem disease, non-elective procedure indication and non-transfemoral access indication.

For peer review only - http://bmjopen.bmj.com/site/about/guidelines.xhtml

Long-term survival was assessed non-parametrically using Kaplan-Meier plots and strata were compared using the log-rank test. Multivariable adjustment was performed using Cox proportional hazards models, using the same variables as above to adjust the frailty indication hazard ratio (HR) for measured confounding. To account for non-proportional hazards, time was split into strata of 0-180 days, 180-365 days and >365 days, with frailty-indication-by-time-indicator interactions included in the Cox proportional hazards models.

The effect on predictive performance of adding CSHA-estimated frailty, KATZ ADL dependency, or physician-estimated poor mobility into the existing TAVI CPMs (FRANCE-2, OBSERVANT and ACC) was considered using model updating techniques [25–27]. Mathematical details of model updating techniques are given in the supplementary methods. In short, CSHA, KATZ, and poor mobility were added (both separately and sequentially for each measure) into an existing CPM by fitting a logistic regression model of 30-day mortality, with both the linear predictor of the considered TAVI CPM and at least one of the frailty measures as covariates. The likelihood ratio test (LRT) was used to test for significant improvement in model fit before and after model updating. Additionally, the area under the receiver operating characteristic curve (AUC) was calculated for each model and compared using a DeLong comparison [28]. Finally, intra-class correlation coefficient (ICC) estimates and their 95% CI were calculated based on an absolute-agreement, two-way random-effects model to indicate the risk-prediction agreement between the TAVI CPMs both before and after the addition of CSHA, KATZ, or poor mobility.

R version 3.4.0 [29] was used for all statistical analyses. Graphical plots were made using the ggplot2 package [30] and the mice package was used for the multiple imputation [31].

Patient and Public Involvement

No patients were involved in the design or conducting of this study.

For peer review only - http://bmjopen.bmj.com/site/about/guidelines.xhtml

Results

Between January 2013 and December 2014, n=3073 patients underwent a TAVI procedure in England and Wales. After excluding 104 patients with missing life-status and 345 patients with any missing frailty measure, 2624 patients were analysed in this study. Due to the relative high proportion of exclusions (14.6%), we examined baseline characteristics of included patients compared with those excluded (**Supplementary Table 1**). Most of the baseline characteristics were similar between the study group and the excluded group, with the exception of access route, proportion of calcified aorta and NYHA≥ III.

A total of 1043 patients (39.7%) had CSHA-estimated frailty (**Supplementary Figure 1**), 846 patients (32.2%) had ADL dependency (KATZ<6) (**Supplementary Figure 2**), and 591 patients (22.5%) had physician-estimated poor mobility. **Figure 1** shows a Venn diagram of the overlap in classification across the three measures. The central segment of the Venn diagram (n=297) is the group defined as "Severely Frail" by the composite score, with the other segments (n=1169) showing the "Moderately Frail" group. The disagreement in **Figure 1** may relate to both the relative imprecision and subjectivity of each frailty measure and that the different measures assess disparate aspects of frailty.

Baseline characteristics and the proportion of missing data for the whole cohort are presented in **Table 2**. Comparisons of baseline characteristics across CSHA-estimated frailty, KATZ ADL dependency and physician-estimated poor mobility groups are given in **Supplementary Table 2**, **Supplementary Table 3** and **Supplementary Table 4**, respectively. CSHA-estimated frail patients were more likely female (p<0.001), with higher proportions of NYHA≥III (p<0.001), LVEF<50% (p=0.001) and non-elective procedures (p<0.001), but with lower numbers of CSHA-estimated frail patients having a previous cardiac surgery (p<0.001). Similar differences were observed between KATZ ADL

For peer review only - http://bmjopen.bmj.com/site/about/guidelines.xhtml

For peer review only

dependency (**Supplementary Table 3**) and between physician-estimated poor mobility groups (**Supplementary Table 4**).

There was large spatial heterogeneity in the proportion of TAVI procedures conducted in patients estimated as having poor mobility, KATZ<6 or CSHA-estimated frailty (**Supplementary Figure 3**). For example, proportions of physician-estimated poor mobility patients across NHS regions ranged from 6.5% to 42.7%; NHS England North (Lancashire and Greater Manchester) had the highest proportion of patients with KATZ<6 and CSHA-estimated frailty, while NHS England South (Wessex) had the highest proportion of physician-estimated poor mobility patients.

Frailty and Post TAVI Outcomes

Incremental increases in components of CSHA and KATZ were associated with increased crude 30-day mortality rates (**Supplementary Figure 1** and **Supplementary Figure 2**). Specifically, CSHA-estimated frail patients had higher crude 30-day mortality, but this was not significant after multivariable adjustment (**Table 3**). In contrast, physician-estimated poor mobility and KATZ<6 were significantly associated with increased odds of 30-day mortality after multivariable adjustment. All three of the considered frailty measures were independently associated with increased VARC-2 composite early safety endpoint (**Table 3**). Similarly, those defined as frail across all three of CSHA, KATZ and poor mobility (i.e. Severely Frail within the composite score) had significantly higher multivariable adjusted odds of 30-day mortality and composite early safety compared with patients defined as Non-Frail by the composite score (**Supplementary Table 5**).

Overall survival was 95.9%, 85.2% and 76.3% at 30-days, one-year and two-years, respectively. Survival was significantly worse for patients with CSHA-estimated frailty ($p<0.0001$), KATZ<6 ($p<0.0001$), physician-estimated poor mobility ($p<0.001$), and across the composite frailty groups ($p<0.0001$) (**Figure 2**). The multivariable time-dependent Cox

For peer review only - http://bmjopen.bmj.com/site/about/guidelines.xhtml

proportional hazards models indicated that hazards of mortality were significantly higher for those with KATZ<6 or physician-estimated poor mobility within one-year post TAVI (**Table 4**); after one-year, multivariable adjusted hazards were not significantly different for these measures. CSHA-estimated frailty had significantly increased hazards of mortality 0-180 days and >365 days post TAVI, but hazards were not significantly different between these time windows. Quantitatively similar results were found across the composite score frailty groups.

Addition of Frailty in TAVI CPMs

Table 5 gives the discrimination of each TAVI CPM for predicting 30-day mortality both before and after the addition of each frailty measure. The LRT indicated a significant improvement in model fit by adding CSHA-estimated frailty into the FRANCE-2 model (p=0.037), the OBSERVANT model (p=0.020) and the ACC model (p=0.048). Adding KATZ<6 into the TAVI models significantly improved the AUC of both the FRANCE-2 CPM (p=0.047) and the OBSERVANT CPM (p=0.007), with the LRT indicating a significant improvement in model fit for all three TAVI CPMs (p<0.001). Equally, physician-estimated poor mobility significantly improved the fit of all three models (LRT p<0.001) and significantly increased the AUC of the OBSERVANT (p=0.006) and the ACC (p=0.030) CPMs by 7% and 5%, respectively. A forward stepwise selection of all three measures resulted in KATZ<6 and physician-estimated poor mobility being added into each of the existing TAVI CPMs (**Table 5**); that is, CSHA-estimated frailty did not significantly improve the predictive performance of the existing TAVI models after inclusion of KATZ<6 and physician-estimated poor mobility.

Finally, the absolute-agreement ICC between the original versions of each TAVI CPM was 0.39 (95% CI: 0.27, 0.49), which increased to 0.65 (95% CI: 0.63, 0.67), 0.78 (95% CI: 0.76, 0.79) and 0.81 (95% CI: 0.79, 0.82) upon adding CSHA-estimated frailty,

For peer review only - http://bmjopen.bmj.com/site/about/guidelines.xhtml

KATZ<6 or physician-estimated poor mobility, respectively. Thus, the patient-level agreement in risk-prediction between the three TAVI CPMs was significantly improved after adding any of CSHA, KATZ, or poor mobility into the models.

Discussion

The findings from this study can be summarised as follows: 1) KATZ<6 and physician-estimated poor mobility were independently associated with increased 30-day mortality, and all three considered frailty measures were associated with increased VARC-2 composite early safety; 2) patients with KATZ<6 and physician-estimated poor mortality had significantly worse survival up to one-year post TAVI; 3) the addition of KATZ and physician-estimated poor mobility into existing TAVI CPMs significantly improved the discrimination and patient-level risk-stratification agreement of the models.

Frailty and Clinical Outcomes Following TAVI

Given that frailty is an indicator of general health status and vulnerability among elderly patients [7], it is naturally under study in the TAVI field. The current study supports and expands the findings from previous studies that have shown associations between frailty and poor TAVI outcomes [8–13,32–34]. For instance, a previous analysis of a single UK centre showed that poor mobility strongly predicted survival following TAVI [10]; the current study supports this finding at the national level, in showing that the odds of 30-day mortality in those with physician-estimated poor mobility were 2.15 times higher compared with normal mobility. Similarly, a multicentre investigation of the Japanese registry found that a semi-quantitative clinical frailty scale was associated with increased 30-day and one-year mortality [12]. Together, the current and previous results suggest that frailty/disability is an indicator of worse outcomes post TAVI, despite the heterogeneity in frailty measures used across the existing evidence base. AHA/ACC guidelines currently indicate that KATZ, gait

For peer review only - http://bmjopen.bmj.com/site/about/guidelines.xhtml

speed and grip strength should be used in the assessment of surgical risk [35], with the emerging data suggesting that similar recommendations should be made for TAVI risk assessment and prediction.

However, implementing this emerging evidence may prove difficult without consensus regarding the most clinically useful measure of frailty in TAVI patients. By comparing the predictive performance of 7 different frailty scales, Afilalo et al. recently recommended that the “Essential Frailty Toolset”, which includes multiple domains of frailty (motor skills, cognition, and nutritional/physiological factors), be used in TAVI patients [11]. Data on such frailty-domains were unavailable for the current study, with the three subjective measures collected within the UK TAVI registry mainly utilising measurements of ADL. Nonetheless, it is conceivable that assessment of ADL (e.g. KATZ) could provide a simple mechanism of informing part of the risk assessment process for TAVI, until more objective frailty scales are routinely collected. For instance, a previous multicentre study by Alfredsson et al. showed that an objective measure of gait speed was associated with 30-day mortality following TAVI [32], but such measures are rarely recorded in national registries, which would currently limit their use to aid risk prediction/stratification in TAVI patients.

Moreover, outcomes after TAVI should consider both mortality and quality of life in the context of the elderly patients that predominantly undergo TAVI. Previous work has shown associations between frailty and quality of life [36] and a contemporary TAVI CPM that was derived to predict poor outcome (defined as mortality and/or reduced quality of life) included measures of frailty, functional status and cognitive decline [37,38]. Within the current study, indicators of quality of life were not available, which should be noted as a limitation of the study. Consequently, further studies are needed to investigate the effect of frailty on endpoints such as quality of life or hospital re-admission, which are increasingly used as a measure of futility in TAVI patients.

For peer review only - http://bmjopen.bmj.com/site/about/guidelines.xhtml

Prognostic Utility of Frailty

Prognostic prediction for TAVI is an ongoing research area, with existing TAVI CPMs reporting only moderate performance when validated [4–6,16]. We found that adding indicators of dependency in ADL (KATZ<6) or physician-estimated poor mobility significantly improved the predictive performance and patient-level risk-stratification agreement of the TAVI models. This supports previous work that has demonstrated improvements in predictive performance of the EuroSCORE and STS models through the addition of frailty measures [11,12,39,40]. Consequently, the heart team should consider frailty measures in addition to the multiple comorbidities that are reflected in existing risk scores, preferably by the inclusion of frailty directly into the risk prediction. We recommend that future iterations of existing and new TAVI risk models should include markers of frailty/disability. Arguably, one should regard frailty as a spectrum, rather than a binary phenomenon; however, the sample size of the current study restricted our ability to sub-classify KATZ and CSHA into their individual components. Moreover, the marginal improvement in predictive demonstrated after the inclusion of frailty/disability measures into existing TAVI CPMs, suggests that these measures cannot overcome the need to discover novel risk factors in this patient cohort, or the need to predict endpoints other than mortality (e.g. readmission or quality of life).

Moreover, the quantification of TAVI risk by using subjective measures of frailty could be problematic in situations of low inter-rater reliability. The current study demonstrated large spatial variability in the proportion of TAVI procedures conducted on 'frail' patients, but the subjectivity of CSHA and KATZ means that we were unable to separate genuine spatial differences from systematic variation in how different teams define/record these measures. This provides further indication for the need to refine the

For peer review only - http://bmjopen.bmj.com/site/about/guidelines.xhtml

assessment of frailty in TAVI patients, since the current methods were derived for different purposes.

Study Limitations

The strength of this study is that it is a large, contemporary study, which has investigated the impact of frailty on outcomes and mortality prediction post TAVI from a national perspective. However, several limitations need to be considered. Firstly, only three subjective frailty measures were available, with objective frailty tests such as 6-min walk distance, grip strength or gait speed not recorded. Secondly, unmeasured confounders inherent in most observational studies potentially influence conclusions. Thirdly, given that we could only analyse TAVI patients in 2013/14, this retrospective study was underpowered to investigate endpoints with small event rates such as bleeding or myocardial infarction. As the volume of frailty data increases, future studies will be able to investigate such outcomes. Fourthly, the current study had a significant proportion of patients who were removed due to missing frailty measures and/or missing life-status, which could potentially bias the results. However, the proportion of missing data in other variables was low and we implemented a multiple imputation procedure. Finally, given that this was a retrospective analysis of a national TAVI registry, we could not examine frailty in untreated aortic stenosis patients, and neither could we compare the outcomes with patients treated through surgical aortic valve replacement. Thus, associations between frailty and the propensity for conservative treatment could not be explored, with corresponding potential for selection bias. Specifically, the analysis highlights potential measures to predict the expected outcomes after TAVI, but not the expected outcomes without TAVI.

For peer review only - http://bmjopen.bmj.com/site/about/guidelines.xhtml

Conclusions

Frailty and disability, as estimated by KATZ, CSHA and poor mobility, was significantly associated with mortality and morbidity after TAVI. The predictive performance and patient-level risk-stratification agreement was significantly improved by updating existing TAVI CPMs to include measures of frailty/ ADL dependency. Hence, physician-estimated frailty/disability measures could aid TAVI risk stratification, until scales that are more objective are routinely collected.

Acknowledgements

We would like to acknowledge all participating centres for collecting the data and the National Institute for Cardiovascular Outcomes Research (NICOR) for provided the UK TAVI registry extract for this study.

Funding Statement

This research was funded by the Medical Research Council, through the Health e-Research Centre, University of Manchester [MR/K006665/1] and the North Staffordshire Heart Committee.

Competing Interests Statement

The authors have no conflict of interest.

Author Contributions

GPM, MS and MAM made substantial contributions to the concept of the work in addition to performing the analysis. GPM drafted the initial version of the manuscript. GPM, MS, PFL, MAB, MG, JT, SR, UK, IB, and MAM interpreted the results, revised the paper critically for

For peer review only - http://bmjopen.bmj.com/site/about/guidelines.xhtml

For peer review only

important intellectual content and approved the final version of the paper. All authors agreed to be accountable for all aspects of the work.

Ethics Approval

The National Institute for Cardiovascular Outcomes Research (NICOR) which includes the UK TAVI registry has support under section 251 of the National Health Service Act 2006 to use patient information for medical research without informed consent. Further ethical approval was not required under current National Health Service research governance arrangements, as all data analysed in the study was pseudonymised and contained no patient identifiable information.

Data Sharing

The data that this research was conducted on is available from the National Institute for Cardiovascular Outcomes Research (NICOR) after undertaking an application process.

For peer review only

For peer review only - http://bmjopen.bmj.com/site/about/guidelines.xhtml

References

1 Leon M, Smith C, Mack M, *et al.* Transcatheter aortic-valve implantation for aortic stenosis in patients who cannot undergo surgery. *N Engl J Med* 2010;**363**:1597–607.

2 Smith C, Leon M, Mack M, *et al.* Transcatheter versus surgical aortic-valve replacement in high-risk patients. *N Engl J Med* 2011;**364**:2187–98.

3 Leon MB, Smith CR, Mack MJ, *et al.* Transcatheter or Surgical Aortic-Valve Replacement in Intermediate-Risk Patients. *N Engl J Med* 2016;**374**:1609–20. doi:10.1056/NEJMoa1514616

4 Iung B, Laouénan C, Himbert D, *et al.* Predictive factors of early mortality after transcatheter aortic valve implantation: individual risk assessment using a simple score. *Heart* 2014;**100**:1016–23. doi:10.1136/heartjnl-2013-305314

5 Capodanno D, Barbanti M, Tamburino C, *et al.* A simple risk tool (the OBSERVANT score) for prediction of 30-day mortality after transcatheter aortic valve replacement. *Am J Cardiol* 2014;**113**:1851–8. doi:10.1016/j.amjcard.2014.03.014

6 Edwards FH, Cohen DJ, O'Brien SM, *et al.* Development and Validation of a Risk Prediction Model for In-Hospital Mortality After Transcatheter Aortic Valve Replacement. *JAMA Cardiol* 2016;**1**:46. doi:10.1001/jamacardio.2015.0326

7 Fried LP, Tangen CM, Walston J, *et al.* Frailty in Older Adults: Evidence for a Phenotype. *Journals Gerontol Ser A Biol Sci Med Sci* 2001;**56**:M146–57. doi:10.1093/gerona/56.3.M146

8 Puls M, Sobisiak B, Bleckmann A, *et al.* Impact of frailty on short- and long-term morbidity and mortality after transcatheter aortic valve implantation: risk assessment by Katz Index of activities of daily living. *EuroIntervention* 2014;**10**:609–19. doi:10.4244/EIJY14M08_03

For peer review only

For peer review only - http://bmjopen.bmj.com/site/about/guidelines.xhtml

9 Rodés-Cabau J, Webb JG, Cheung A, *et al.* Long-term outcomes after transcatheter aortic valve implantation: Insights on prognostic factors and valve durability from the Canadian Multicenter Experience. *J Am Coll Cardiol* 2012;**60**:1864–75. doi:10.1016/j.jacc.2012.08.960

10 Cockburn J, Singh MS, Rafi NHM, *et al.* Poor mobility predicts adverse outcome better than other frailty indices in patients undergoing transcatheter aortic valve implantation. *Catheter Cardiovasc Interv* 2015;**86**:1271–7. doi:10.1002/ccd.25991

11 Afilalo J, Lauck S, Kim DH, *et al.* Frailty in Older Adults Undergoing Aortic Valve Replacement: The FRAILTY-AVR Study. *J Am Coll Cardiol* 2017;**70**:689–700. doi:10.1016/j.jacc.2017.06.024

12 Shimura T, Yamamoto M, Kano S, *et al.* Impact of the Clinical Frailty Scale on Outcomes After Transcatheter Aortic Valve Replacement. *Circulation* 2017;**135**:2013–24. doi:10.1161/CIRCULATIONAHA.116.025630

13 Kleczynski P, Dziewierz A, Bagienski M, *et al.* Impact of frailty on mortality after transcatheter aortic valve implantation. *Am Heart J* 2017;**185**:52–8. doi:10.1016/j.ahj.2016.12.005

14 Kappetein AP, Head SJ, Genereux P, *et al.* Updated standardized endpoint definitions for transcatheter aortic valve implantation: the Valve Academic Research Consortium-2 consensus document. *Eur Heart J* 2012;**33**:2403–18. doi:10.1093/eurheartj/ehs255

15 Durand E, Borz B, Godin M, *et al.* Performance analysis of EuroSCORE II compared to the original logistic EuroSCORE and STS scores for predicting 30-day mortality after transcatheter aortic valve replacement. *Am J Cardiol* 2013;**111**:891–7. doi:10.1016/j.amjcard.2012.11.056

16 Martin GP, Sperrin M, Ludman PF, *et al.* Inadequacy of existing clinical prediction models for predicting mortality after transcatheter aortic valve implantation. *Am Heart*

For peer review only - http://bmjopen.bmj.com/site/about/guidelines.xhtml

J 2017;**184**:97–105. doi:10.1016/j.ahj.2016.10.020

17 Halkin A, Steinvil A, Witberg G, *et al.* Mortality prediction following transcatheter aortic valve replacement: A quantitative comparison of risk scores derived from populations treated with either surgical or percutaneous aortic valve replacement. The Israeli TAVR Registry Risk Model Accuracy A. *Int J Cardiol* 2016;**215**:227–31. doi:10.1016/j.ijcard.2016.04.038

18 Ludman PF. The UK transcatheter aortic valve implantation registry; one of the suite of registries hosted by the National Institute for Cardiovascular Outcomes Research (NICOR). *Heart* 2012;**98**:1787–9. doi:10.1136/heartjnl-2012-302534

19 Rockwood K, Song X, MacKnight C, *et al.* A global clinical measure of fitness and frailty in elderly people. *C Can Med Assoc J* 2005;**173**:489–95. doi:10.1503/cmaj.050051

20 Katz S. Studies of Illness in the Aged. The Index of ADL: A Standardized Measure of Biological and Psychosocial Function. *JAMA* 1963;**185**:914. doi:10.1001/jama.1963.03060120024016

21 Nashef SAM, Roques F, Sharples LD, *et al.* EuroSCORE II. *Eur J Cardio-Thoracic Surg* 2012;**41**:734–45. doi:10.1093/ejcts/ezs043

22 Rubin DB. *Multiple Imputation for Nonresponse in Surveys*. John Wiley & Sons 1987.

23 Sterne JAC, White IR, Carlin JB, *et al.* Multiple imputation for missing data in epidemiological and clinical research: potential and pitfalls. *BMJ* 2009;**338**:b2393–b2393. doi:10.1136/bmj.b2393

24 von Hippel PT. Regression with Missing YS: An Improved Strategy For Analyzing Multiply Imputed Data. *Sociol Methodol* 2007;**37**:83–117. doi:10.1111/j.1467-9531.2007.00180.x

25 Steyerberg EW, Borsboom GJJM, van Houwelingen HC, *et al.* Validation and

For peer review only - http://bmjopen.bmj.com/site/about/guidelines.xhtml

updating of predictive logistic regression models: a study on sample size and shrinkage. *Stat Med* 2004;**23**:2567–86. doi:10.1002/sim.1844

26 Janssen KJM, Moons KGM, Kalkman CJ, *et al.* Updating methods improved the performance of a clinical prediction model in new patients. *J Clin Epidemiol* 2008;**61**:76–86. doi:10.1016/j.jclinepi.2007.04.018

27 Steyerberg EW. *Clinical Prediction Models*. Springer New York 2009. doi:10.1007/978-0-387-77244-8

28 DeLong ER, DeLong DM, Clarke-Pearson DL. Comparing the Areas under Two or More Correlated Receiver Operating Characteristic Curves: A Nonparametric Approach. *Biometrics* 1988;**44**:837. doi:10.2307/2531595

29 R Core Team R. R: A Language and Environment for Statistical Computing. R Found. Stat. Comput. 2017. doi:10.1007/978-3-540-74686-7

30 Wickham H. *ggplot2: elegant graphics for data analysis*. Springer New York 2009.

31 Buuren S Van, Groothuis-Oudshoorn K. mice: Multivariate Imputation by Chained Equations in R. *J Stat Softw* 2011;**45**:1–67.http://www.jstatsoft.org/v45/i03/

32 Alfredsson J, Stebbins A, Brennan JM, *et al.* Gait speed predicts 30-day mortality after transcatheter aortic valve replacement: Results from the Society of Thoracic Surgeons/American College of Cardiology Transcatheter Valve Therapy Registry. *Circulation* 2016;**133**:1351–9. doi:10.1161/CIRCULATIONAHA.115.020279

33 Green P, Woglom AE, Genereux P, *et al.* The impact of frailty status on survival after transcatheter aortic valve replacement in older adults with severe aortic stenosis: A single-center experience. *JACC Cardiovasc Interv* 2012;**5**:974–81. doi:10.1016/j.jcin.2012.06.011

34 Okoh AK, Chauhan D, Kang N, *et al.* The impact of frailty status on clinical and functional outcomes after transcatheter aortic valve replacement in nonagenarians with

For peer review only

For peer review only - http://bmjopen.bmj.com/site/about/guidelines.xhtml

severe aortic stenosis. *Catheter Cardiovasc Interv* 2017;**90**:1000–6. doi:10.1002/ccd.27083

35 Nishimura RA, Otto CM, Bonow RO, *et al.* 2014 AHA/ACC Guideline for the Management of Patients With Valvular Heart Disease. *Circulation* 2014;**129**:e521 LP-e643.http://circ.ahajournals.org/content/129/23/e521.abstract

36 Green P, Arnold S V., Cohen DJ, *et al.* Relation of Frailty to Outcomes After Transcatheter Aortic Valve Replacement (from the PARTNER Trial). *Am J Cardiol* 2015;**116**:264–9. doi:10.1016/j.amjcard.2015.03.061

37 Arnold S V., Reynolds MR, Lei Y, *et al.* Predictors of poor outcomes after transcatheter aortic valve replacement results from the PARTNER (Placement of Aortic Transcatheter Valve) trial. *Circulation* 2014;**129**:2682–90. doi:10.1161/CIRCULATIONAHA.113.007477

38 Arnold S V., Afilalo J, Spertus JA, *et al.* Prediction of Poor Outcome After Transcatheter Aortic Valve Replacement. *J Am Coll Cardiol* 2016;**68**:1868–77. doi:10.1016/j.jacc.2016.07.762

39 Rogers T, Alraies MC, Pacha HM, *et al.* Clinical Frailty as an Outcome Predictor After Transcatheter Aortic Valve Implantation. *Am J Cardiol* Published Online First: January 2018. doi:10.1016/j.amjcard.2017.12.035

40 Schoenenberger AW, Moser A, Bertschi D, *et al.* Improvement of Risk Prediction After Transcatheter Aortic Valve Replacement by Combining Frailty With Conventional Risk Scores. *JACC Cardiovasc Interv* 2018;**11**:395–403. doi:10.1016/j.jcin.2017.11.012

For peer review only

For peer review only - http://bmjopen.bmj.com/site/about/guidelines.xhtml

Figure Legends

Figure 1: Venn diagram showing the overlap in the different definitions of frailty/disability across CSHA-estimated frailty, KATZ<6 and physician-estimated poor mobility; the sizes of each segment are proportional to the segment sample sizes.

Figure 2: Kaplan-Meier plots across CSHA-estimated frailty (top-left), KATZ activities of daily living dependency (top-right), physician-estimated poor mobility (bottom-left), and the composite frailty score (bottom-right). Definitions are described in Table 1.

For peer review only

Tables

Table 1. Frailty grouping definitions by each frailty/disability measure.

Frailty/disability Measure	Scoring System	Frailty definition used in this analysis
CSHA*	A physician-estimated frailty score, based on the following options: 1. Very fit; 2. Well; 3. Well with treated comorbid disease; 4. Apparently vulnerable; 5. Mildly frail with limited dependence for activities of daily living (ADL); 6. Moderately frail requiring help with ADL; 7. Severely frail being completely dependent.	CSHA options of 5, 6, and 7 were used to define CSHA Frail patients; options 1, 2, 3 and 4 were classed as CSHA Non-Frail.
KATZ*	0 to 6-point scale assessing dependency in the following ADL: 1. Bathing; 2. Dressing; 3. Toileting; 4. Transferring; 5. Continence; 6. Feeding.	Any patient with a KATZ Score < 6 points compared with those with KATZ=6.
Poor Mobility	A physician-estimated indication of any severe impairment of mobility that is secondary to musculoskeletal or neurological dysfunction.	Any patient defined as having poor mobility compared with those defined as having normal mobility.
Composite Score	Combination of CSHA, KATZ and poor mobility.	Defined as the following:<br>(i) Non-Frail if a patient is estimated as not frail by CSHA, KATZ and poor mobility;<br>(ii) Moderately Frail if a patient is estimated as frail by at least one (but not all) considered frailty

For peer review only - http://bmjopen.bmj.com/site/about/guidelines.xhtml

		measures;<br>(iii) Severely Frail if a patient is classed as frail across all three of CSHA, KATZ and poor mobility.

**: dichotomising of CSHA and KATZ into two groups was based on the median level observed in the TAVI registry (see Supplementary Figures 1 and 2 for the patient distribution of these scores).*

For peer review only

For peer review only - http://bmjopen.bmj.com/site/about/guidelines.xhtml

Table 2. Baseline characteristics and number of missing data within the whole cohort.

**Variable**	**Whole Cohort (n=2624)**	**Missing, n (%)**
Age, mean (SD)	81.2 (7.58)	0 (0.00%)
Female, n (%)	1192 (45.4)	1 (0.04%)
Diabetic, n (%)	641 (24.4)	2 (0.08%)
Smoker, n (%)	1316 (50.2)	45 (1.71%)
Creatinine, mean (SD)	110.9 (60.5)	6 (0.23%)
Renal Failure *, n (%)	143 (5.45)	10 (0.38%)
Previous MI, n (%)	558 (21.3)	2 (0.08%)
Pulmonary Disease, n (%)	791 (30.1)	3 (0.11%)
Neurological Disease, n (%)	441 (16.8)	1 (0.04%)
Extracardiac Arteriopathy, n (%)	555 (21.2)	5 (0.19%)
Calcification of Ascending Aorta, % (n)	378 (14.4)	22 (0.84%)
Atrial Fibrillation, n (%)	700 (26.7)	23 (0.88%)
Previous Cardiac Surgery, n (%)	799 (30.4)	5 (0.19%)
Previous PCI, n (%)	524 (20.0)	2 (0.08%)
Height, mean (SD)	1.64 (0.10)	19 (0.72%)
Weight, mean (SD)	75.0 (17.1)	18 (0.69%)
CCS Class 4, n (%)	24 (0.91)	3 (0.11%)
NYHA ≥ III, n (%)	1985 (75.6)	6 (0.23%)
Aortic Valve Area, mean (SD)	0.69 (0.23)	147 (5.60%)
Aortic Valve Peak Gradient, mean (SD)	71.2 (26.2)	129 (4.92%)
LVEF < 50%, n (%)	948 (36.1)	12 (0.46%)
One or more diseased vessels, n (%)	1058 (40.3)	47 (1.79%)
Left Main Stem Disease, n (%)	103 (3.93)	75 (2.86%)
Non-elective Procedure, n (%)	365 (13.9)	2 (0.08%)
Access Site		
Transfemoral, n (%)	2127 (81.1)	3 (0.11%)
Transapical, n (%)	249 (9.49)	3 (0.11%)
Subclavian, n (%)	85 (3.24)	3 (0.11%)
Other, n (%)	160 (6.10)	3 (0.11%)

** Defined as creatinine >200µmol/l or dialysis for renal failure. LVEF: Left Ventricular Ejection Fraction, MI: Myocardial Infarction, NYHA: New York Heart Association, PCI: Percutaneous coronary intervention.*

For peer review only - http://bmjopen.bmj.com/site/about/guidelines.xhtml

Table 3. Short-term outcomes across CSHA-estimated frailty, KATZ ADL dependency and physician-estimated poor mobility groups.

**Outcome**	**CSHA-estimated frail (n=1043)**	**CSHA-estimated non-frail (n=1581)**	**Univariable OR (95% CI)***	**Multivariable OR (95% CI)***
30-day mortality	57/1043 (5.47%)	51/1581 (3.23%)	**1.73 (1.18, 2.55)**	1.46 (0.96, 2.23)
Early Safety	187/1014 (18.44%)	190/1540 (12.34%)	**1.61 (1.29, 2.00)**	**1.45 (1.14, 1.84)**
**Outcome**	**KATZ<6 (n=846)**	**KATZ=6 (n=1778)**	**Univariable OR (95% CI)***	**Multivariable OR (95% CI)***
30-day mortality	58/846 (6.86%)	50/1778 (2.81%)	**2.54 (1.73, 3.75)**	**2.10 (1.39, 3.15)**
Early Safety	150/827 (18.14%)	227/1727 (13.14%)	**1.46 (1.17, 1.83)**	**1.28 (1.01, 1.63)**
**Outcome**	**Poor mobility (n=591)**	**Normal mobility (n=2033)**	**Univariable OR (95% CI)***	**Multivariable OR (95% CI)***
30-day mortality	46/591 (7.78%)	62/2033 (3.05%)	**2.68 (1.81, 3.98)**	**2.15 (1.41, 3.28)**
Early Safety	116/571 (20.32%)	261/1983 (13.16%)	**1.68 (1.32, 2.14)**	**1.45 (1.12, 1.88)**

*: *Bold items indicate significant results. CI: Confidence Interval, OR: Odds Ratio*

For peer review only - http://bmjopen.bmj.com/site/about/guidelines.xhtml

Table 4. Time dependent Cox proportional hazards models per frailty indicator.

Frailty-by-time interaction	Univariable HR (95% CI)*	Multivariable HR (95% CI)*
CSHA-estimated Frail		
0-180 days	**1.56 (1.22, 1.98)**	**1.29 (1.00, 1.66)**
180-365 days	**1.63 (1.12, 2.38)**	1.37 (0.94, 2.01)
>365 days	**1.85 (1.32, 2.60)**	**1.61 (1.14, 2.29)**
KATZ<6		
0-180 days	**1.71 (1.34, 2.18)**	**1.55 (1.21, 1.99)**
180-365 days	**1.86 (1.28, 2.71)**	**1.74 (1.19, 2.55)**
>365 days	1.25 (0.88, 1.78)	1.23 (0.86, 1.75)
Physician-estimated poor Mobility		
0-180 days	**1.74 (1.34, 2.25)**	**1.50 (1.15, 1.96)**
180-365 days	**2.06 (1.39, 3.06)**	**1.84 (1.24, 2.75)**
>365 days	**1.50 (1.03, 2.20)**	1.36 (0.92, 2.00)

**: Bold items indicate significant results.HR: hazard ratio.*

For peer review only

For peer review only - http://bmjopen.bmj.com/site/about/guidelines.xhtml

Table 5. Discrimination of each TAVI CPM at predicting 30-day mortality before and after the addition of each frailty measure.

Model	AUC (95% CI)	p-value*
**FRANCE-2**		
Original	0.62 (0.57, 0.68)	N/A
Updated with CSHA only	0.64 (0.58, 0.69)	0.412
Updated with KATZ only	0.67 (0.61, 0.72)	0.047
Updated with Poor Mobility only	0.67 (0.62, 0.72)	0.058
Updated with stepwise selection †	0.68 (0.63, 0.73)	0.025
**OBSERVANT**		
Original	0.56 (0.50, 0.62)	N/A
Updated with CSHA only	0.59 (0.53, 0.65)	0.129
Updated with KATZ only	0.62 (0.56, 0.68)	0.007
Updated with Poor Mobility only	0.63 (0.57, 0.69)	0.006
Updated with stepwise selection †	0.64 (0.58, 0.71)	0.002
**ACC**		
Original	0.63 (0.58, 0.68)	N/A
Updated with CSHA only	0.64 (0.59, 0.69)	0.605
Updated with KATZ only	0.67 (0.61, 0.72)	0.069
Updated with Poor Mobility only	0.68 (0.63, 0.73)	0.030
Updated with stepwise selection †	0.68 (0.63, 0.74)	0.036

** the DeLong p-value compares the AUC of predicting 30-day mortality using the original TAVI CPM with that using each corresponding updated version. †: forward selection across the three frailty measures resulted in KATZ<6 and physician-estimated poor mobility being added into each existing TAVI CPM.*

For peer review only - http://bmjopen.bmj.com/site/about/guidelines.xhtml

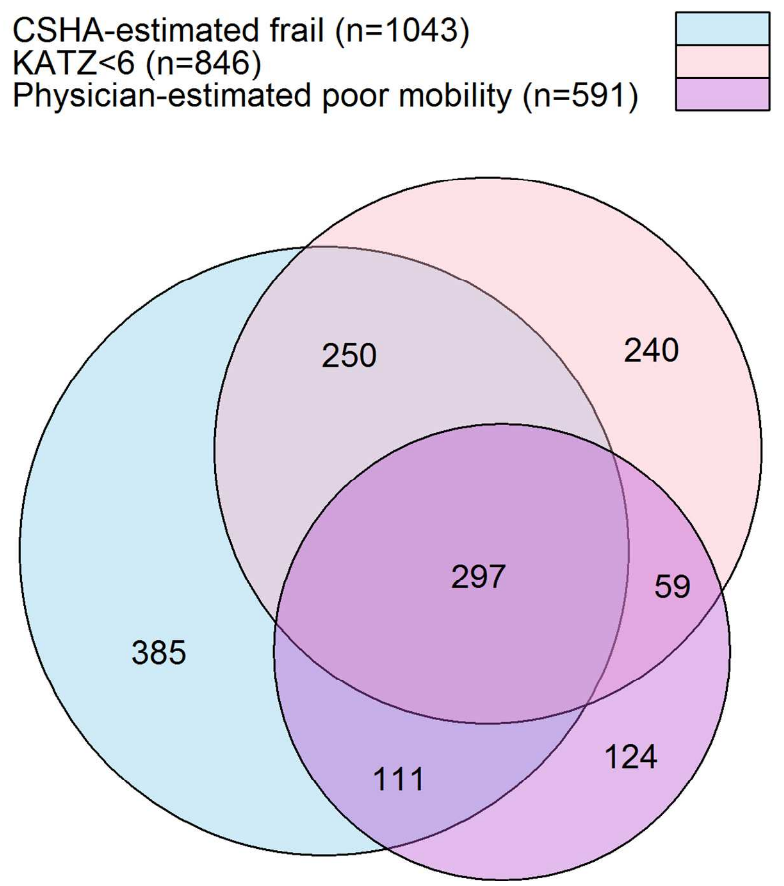

Venn diagram showing the overlap in the different definitions of frailty/disability across CSHA-estimated frailty, KATZ<6 and physician-estimated poor mobility; the sizes of each segment are proportional to the segment sample sizes.

127x127mm (300 x 300 DPI)

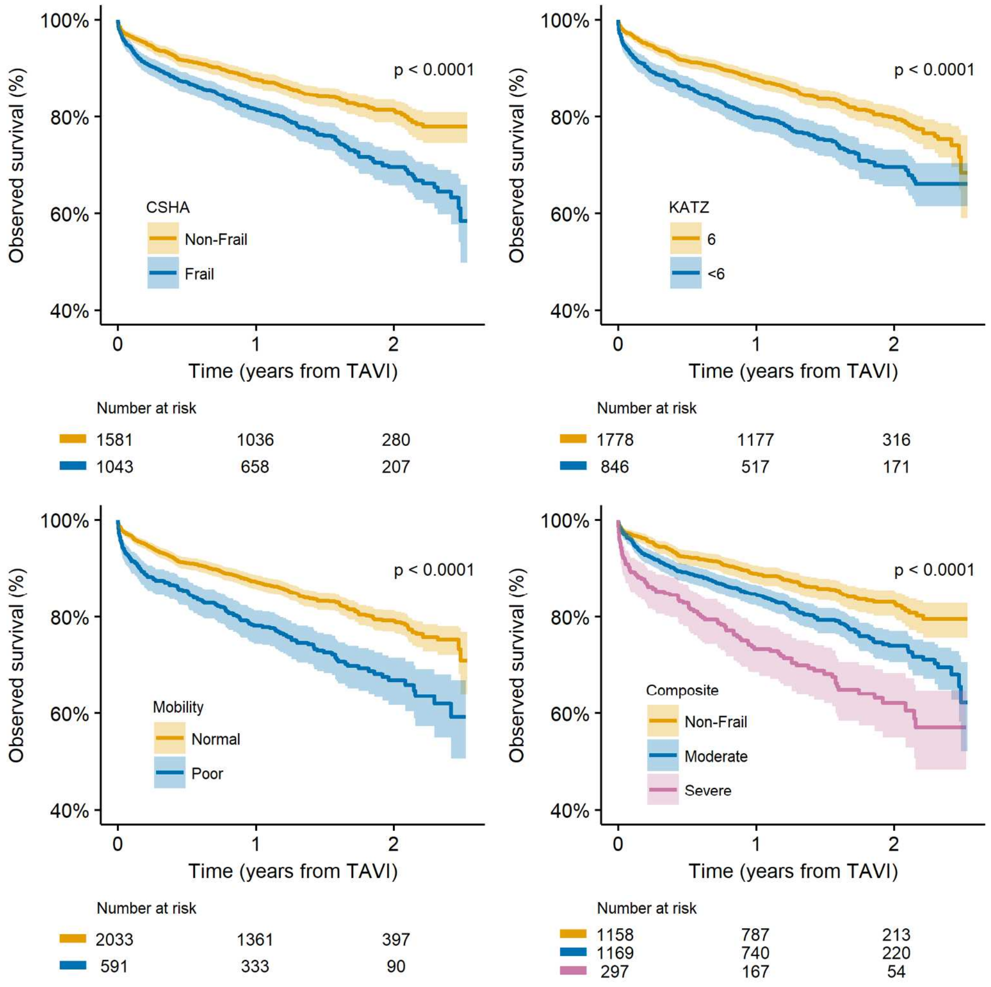

Kaplan-Meier plots across CSHA-estimated frailty (top-left), KATZ activities of daily living dependency (top-right), physician-estimated poor mobility (bottom-left), and the composite frailty score (bottom-right). Definitions are described in Table 1.

177x177mm (300 x 300 DPI)

Supplementary Material

Do frailty measures improve prediction of mortality and morbidity following transcatheter aortic valve implantation? An analysis of the UK TAVI registry

Glen P. Martin; Matthew Sperrin; Peter F. Ludman; Mark A. de Belder; Mark Gunning; John Townend; Simon Redwood; Umesh Kadam; Iain Buchan; Mamas A. Mamas

Supplementary Methods

Mathematical Details of Model Updating Techniques

Let P denote the number of predictors that are included in an existing TAVI CPM. Then the jth existing TAVI CPM provides a linear predictor (LP) on the logit scale of the form

$$\text{LP}_{i,j} = \beta_{0,j} + \sum_{p=1}^{P} \beta_{p,j} x_{i,p}$$

for patient i, covariates $x_{i,p}$ for $p \in [1, P]$, and associated coefficients $\beta_{p,j}$.

Then model updating methods, as previously described [1–4], have a hierarchical structure to alter an existing CPM to a new population. This study considered the addition of new variables that were not included in the original TAVI CPMs (i.e. frailty). Specifically, model updating of the jth existing TAVI CPM fits a logistic regression model in the UK TAVI registry with the jth existing LP and a frailty indicator as the only covariates, by modelling

$$\log\left(\frac{\pi_i}{1-\pi_i}\right) = \hat{\alpha}_0 + \hat{\alpha}_1 \text{LP}_{i,j} + \delta_{\text{Frailty}} x_{i,\text{Frailty}} \quad (1)$$

where π_i denotes the predicted event probability for patient i in the UK TAVI registry, $x_{i,\text{Frailty}}$ is either KATZ, CSHA or poor mobility indication, and δ_{Frailty} is the corresponding coefficient estimate. The parameters $\hat{\alpha}_0$ and $\hat{\alpha}_1$ are the calibration intercept and slope, respectively.

This process was repeated for all three of the frailty measures individually and across all of the considered TAVI CPMs. Additionally, to explore the incremental benefit of adding the frailty measures into the TAVI CPMs, we iteratively added each of the frailty measures into Equation (1), under a likelihood ratio test selection criterion until all those frailty measures that significantly improved model fit were selected.

All models were fit in each multiple imputed dataset separately, with the coefficient estimates then pooled according to Rubin's rules. Predictions were then made for each patient in the UK registry, using the pooled coefficient estimates. Subsequent performance estimates (e.g. area under the ROC curve) were made in each imputed dataset, which were then pooled using Rubin's rules.

For peer review only

Supplementary Figures and Tables

Supplementary Figure 1: Distribution of the CSHA frailty score (left panel) and the crude 30-day mortality rate (right panel)

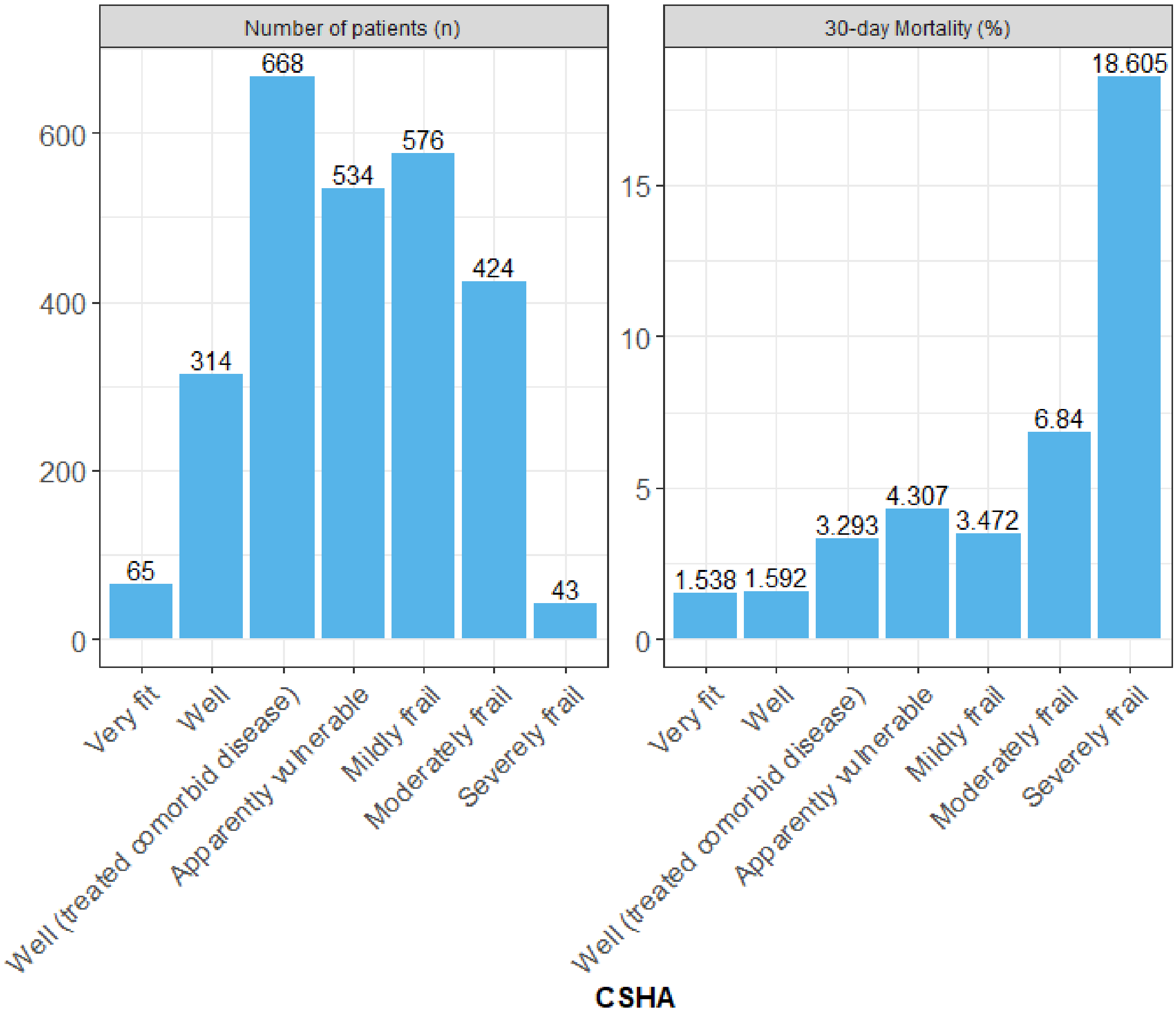

Supplementary Figure 2: Distribution of the KATZ frailty score (left panel) and the crude 30-day mortality rate (right panel)

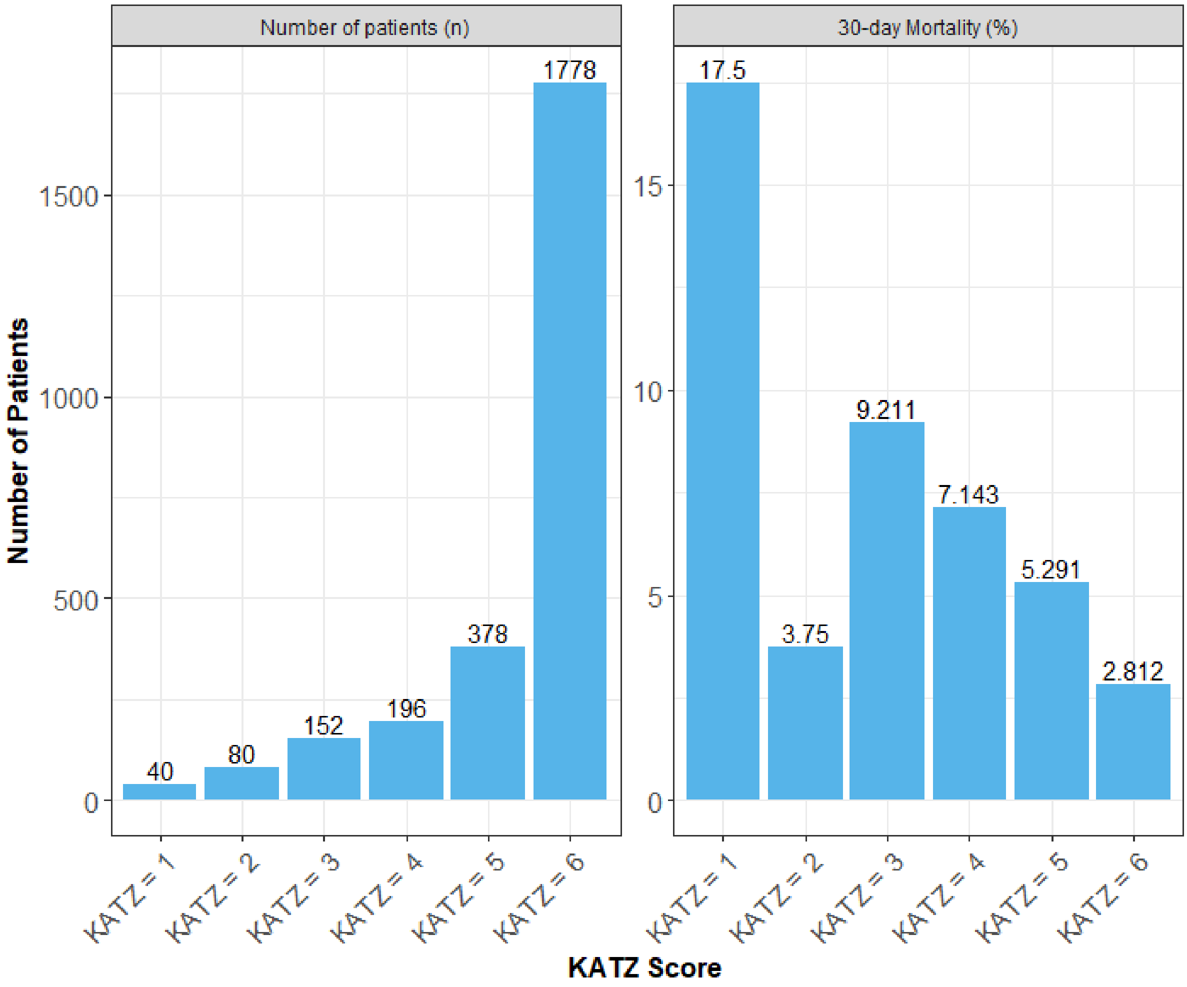

Supplementary Figure 3: Proportion of patients defined as having CSHA-estimated frailty, KATZ activities of daily living dependency, or physician-estimated poor mobility per NHS England region and Wales. The sample size of each region is also depicted.

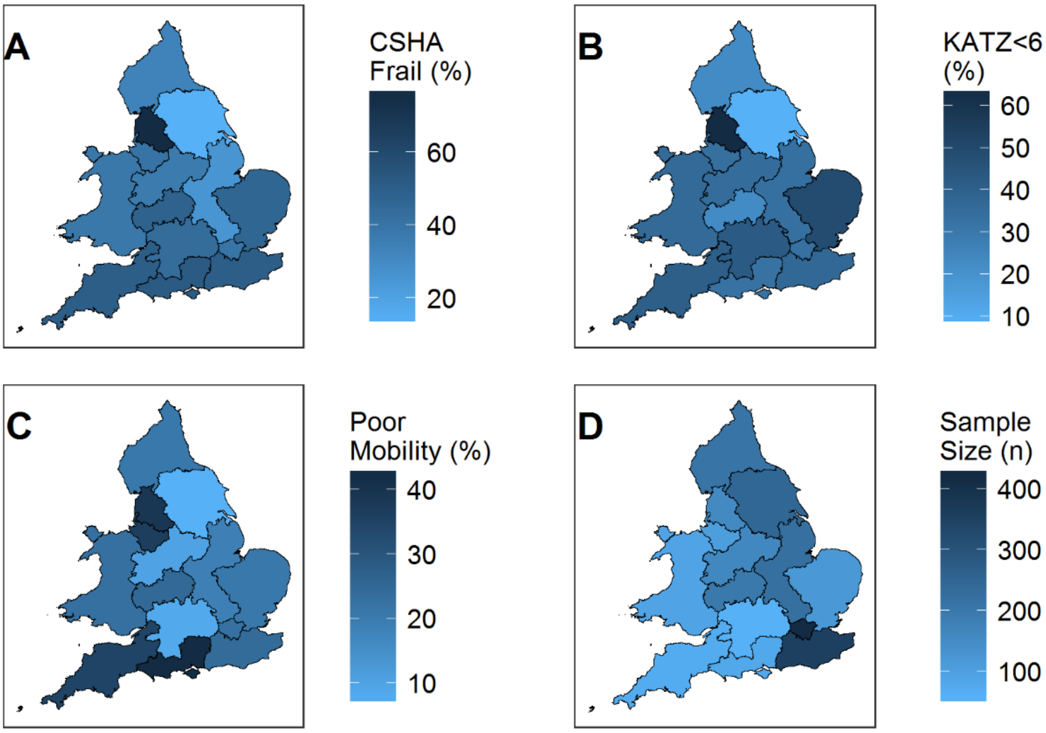

Supplementary Table 1. Baseline characteristics across the patients included and excluded in the analysis.

Variable	Included (n=2624)	Excluded (n=449)	p-value
Age, mean (SD)	81.2 (7.58)	80.6 (7.78)	0.127
Female, n (%)	1192 (45.4)	201 (44.8)	0.807
Diabetic, n (%)	641 (24.4)	97 (21.6)	0.573
Smoker, n (%)	1316 (50.2)	193 (43.0)	0.227
Creatinine, mean (SD)	110.9 (60.5)	111.0 (61.2)	0.962
Renal Failure *, n (%)	143 (5.45)	28 (6.24)	0.270
Previous MI, n (%)	558 (21.3)	96 (21.4)	0.506
Pulmonary Disease, n (%)	791 (30.1)	110 (24.5)	0.115
Neurological Disease, n (%)	441 (16.8)	69 (15.4)	0.932
Extracardiac Arteriopathy, n (%)	555 (21.2)	77 (17.1)	0.227
Calcification of Ascending Aorta, % (n)	378 (14.4)	39 (8.69)	0.005
Atrial Fibrillation, n (%)	700 (26.7)	114 (25.4)	0.837
Previous Cardiac Surgery, n (%)	799 (30.4)	109 (24.3)	0.062
Previous PCI, n (%)	524 (20.0)	86 (19.2)	0.903
Height, mean (SD)	1.64 (0.10)	1.66 (0.10)	0.020
Weight, mean (SD)	75.0 (17.1)	76.2 (17.4)	0.209
CCS Class 4, n (%)	24 (0.91)	7 (1.56)	0.243
NYHA ≥ III, n (%)	1985 (75.6)	347 (77.3)	0.001
Aortic Valve Area, mean (SD)	0.69 (0.23)	0.68 (0.23)	0.468
Aortic Valve Peak Gradient, mean (SD)	71.2 (26.2)	72.9 (24.4)	0.243
LVEF < 50%, n (%)	948 (36.1)	161 (35.9)	0.474
One or more diseased vessels, n (%)	1058 (40.3)	151 (33.6)	0.064
Left Main Stem Disease, n (%)	103 (3.93)	18 (4.01)	0.866
Non-elective Procedure, n (%)	365 (13.9)	62 (13.8)	0.999
Access Site			
Transfemoral, n (%)	2127 (81.1)	334 (74.4)	0.005
Transapical, n (%)	249 (9.49)	75 (16.7)	<0.001
Subclavian, n (%)	85 (3.24)	5 (1.11)	0.022
Other, n (%)	160 (6.10)	30 (6.68)	0.674

** Defined as creatinine >200µmol/l or dialysis for renal failure. LVEF: Left Ventricular Ejection Fraction, MI: Myocardial Infarction, NYHA: New York Heart Association, PCI: Percutaneous coronary intervention.*

For peer review only

Supplementary Table 2. Baseline characteristics across CSHA-estimated frailty groups.

**Variable**	**CSHA-estimated Frail (n=1043)**	**CSHA-estimated Non-Frail (n=1581)**	**p-value**
Age, mean (SD)	82.0 (7.13)	80.7 (7.82)	<0.001
Female, n (%)	525 (50.3)	667 (42.2)	<0.001
Diabetic, n (%)	280 (26.8)	361 (22.8)	0.021
Smoker, n (%)	518 (49.7)	798 (50.5)	0.962
Creatinine, mean (SD)	116.5 (67.0)	107.2 (55.6)	<0.001
Renal Failure *, n (%)	72 (6.90)	71 (4.49)	0.010
Previous MI, n (%)	246 (23.6)	312 (19.7)	0.021
Pulmonary Disease, n (%)	321 (30.8)	470 (29.7)	0.564
Neurological Disease, n (%)	199 (19.1)	242 (15.3)	0.014
Extracardiac Arteriopathy, n (%)	268 (25.7)	287 (18.2)	<0.001
Calcification of Ascending Aorta, % (n)	116 (11.1)	262 (16.6)	<0.001
Atrial Fibrillation, n (%)	296 (28.4)	404 (25.6)	0.138
Previous Cardiac Surgery, n (%)	276 (26.5)	523 (33.1)	<0.001
Previous PCI, n (%)	201 (19.3)	323 (20.4)	0.501
Height, mean (SD)	1.63 (0.10)	1.65 (0.10)	<0.001
Weight, mean (SD)	73.7 (17.5)	75.8 (16.7)	0.003
CCS Class 4, n (%)	12 (1.15)	12 (0.76)	0.412
NYHA ≥ III, n (%)	922 (88.4)	1063 (67.2)	<0.001
Aortic Valve Area, mean (SD)	0.68 (0.22)	0.69 (0.24)	0.092
Aortic Valve Peak Gradient, mean (SD)	67.4 (26.2)	73.7 (25.9)	<0.001
LVEF < 50%, n (%)	416 (39.9)	532 (33.6)	0.001
One or more diseased vessels, n (%)	439 (42.1)	619 (39.2)	0.190
Left Main Stem Disease, n (%)	56 (5.37)	47 (2.97)	0.002
Non-elective Procedure, n (%)	199 (19.1)	166 (10.5)	<0.001
Access Site			
Transfemoral, n (%)	840 (80.5)	1287 (81.4)	0.546
Transapical, n (%)	91 (8.72)	158 (9.99)	0.302
Subclavian, n (%)	34 (3.26)	51 (3.23)	0.999
Other, n (%)	78 (7.48)	82 (5.19)	0.021

** Defined as creatinine >200μmol/l or dialysis for renal failure. LVEF: Left Ventricular Ejection Fraction, MI: Myocardial Infarction, NYHA: New York Heart Association, PCI: Percutaneous coronary intervention.*

For peer review only

Supplementary Table 3. Baseline characteristics across frailty groups defined with KATZ<6.

**Variable**	**KATZ<6 (n=846)**	**KATZ=6 (n=1778)**	**p-value**
Age, mean (SD)	81.5 (7.38)	81.1 (7.67)	0.199
Female, n (%)	417 (49.3)	775 (43.6)	0.007
Diabetic, n (%)	217 (25.7)	424 (23.8)	0.347
Smoker, n (%)	434 (51.3)	882 (49.6)	0.252
Creatinine, mean (SD)	113.6 (69.1)	109.6 (56.0)	0.121
Renal Failure *, n (%)	48 (5.67)	95 (5.34)	0.783
Previous MI, n (%)	198 (23.4)	360 (20.2)	0.075
Pulmonary Disease, n (%)	279 (33.0)	512 (28.8)	0.028
Neurological Disease, n (%)	159 (18.8)	282 (15.9)	0.069
Extracardiac Arteriopathy, n (%)	184 (21.7)	371 (20.9)	0.635
Calcification of Ascending Aorta, % (n)	134 (15.8)	244 (13.7)	0.167
Atrial Fibrillation, n (%)	263 (31.1)	437 (24.6)	<0.001
Previous Cardiac Surgery, n (%)	228 (27.0)	571 (32.1)	0.009
Previous PCI, n (%)	165 (19.5)	359 (20.2)	0.725
Height, mean (SD)	1.64 (0.10)	1.65 (0.10)	0.227
Weight, mean (SD)	75.1 (17.0)	74.9 (17.1)	0.843
CCS Class 4, n (%)	10 (1.18)	14 (0.79)	0.439
NYHA ≥ III, n (%)	677 (80.0)	1308 (73.6)	<0.001
Aortic Valve Area, mean (SD)	0.68 (0.23)	0.69 (0.23)	0.536
Aortic Valve Peak Gradient, mean (SD)	70.4 (24.4)	71.6 (27.1)	0.271
LVEF < 50%, n (%)	356 (42.1)	592 (33.3)	<0.001
One or more diseased vessels, n (%)	352 (41.6)	706 (39.7)	0.416
Left Main Stem Disease, n (%)	36 (4.26)	67 (3.77)	0.655
Non-elective Procedure, n (%)	161 (19.0)	204 (11.5)	<0.001
Access Site			
Transfemoral, n (%)	704 (83.2)	1423 (80.0)	0.070
Transapical, n (%)	76 (8.98)	173 (9.73)	0.581
Subclavian, n (%)	22 (2.60)	63 (3.54)	0.244
Other, n (%)	44 (5.20)	116 (6.52)	0.213

** Defined as creatinine >200µmol/l or dialysis for renal failure. LVEF: Left Ventricular Ejection Fraction, MI: Myocardial Infarction, NYHA: New York Heart Association, PCI: Percutaneous coronary intervention.*

For peer review only

Supplementary Table 4. Baseline characteristics across those with/without physician-estimated poor mobility.

**Variable**	**Poor Mobility (n=591)**	**Normal Mobility (n=2033)**	**p-value**
Age, mean (SD)	81.4 (7.92)	81.1 (7.47)	0.481
Female, n (%)	328 (55.5)	864 (42.5)	<0.001
Diabetic, n (%)	151 (25.5)	490 (24.1)	0.496
Smoker, n (%)	288 (48.7)	1028 (50.6)	0.677
Creatinine, mean (SD)	115.3 (80.3)	109.6 (53.4)	0.045
Renal Failure *, n (%)	42 (7.11)	101 (4.97)	0.055
Previous MI, n (%)	128 (21.7)	430 (21.2)	0.844
Pulmonary Disease, n (%)	206 (34.9)	585 (28.8)	0.004
Neurological Disease, n (%)	112 (19.0)	329 (16.2)	0.129
Extracardiac Arteriopathy, n (%)	165 (27.9)	390 (19.2)	<0.001
Calcification of Ascending Aorta, % (n)	91 (15.4)	287 (14.1)	0.371
Atrial Fibrillation, n (%)	168 (28.4)	532 (26.2)	0.287
Previous Cardiac Surgery, n (%)	131 (22.2)	668 (32.9)	<0.001
Previous PCI, n (%)	93 (15.7)	431 (21.2)	0.004
Height, mean (SD)	1.63 (0.10)	1.65 (0.10)	<0.001
Weight, mean (SD)	74.8 (17.9)	75.0 (16.8)	0.818
CCS Class 4, n (%)	7 (1.18)	17 (0.84)	0.590
NYHA ≥ III, n (%)	489 (82.7)	1496 (73.6)	<0.001
Aortic Valve Area, mean (SD)	0.67 (0.23)	0.69 (0.23)	0.110
Aortic Valve Peak Gradient, mean (SD)	69.3 (25.3)	71.8 (26.5)	0.050
LVEF < 50%, n (%)	224 (37.9)	724 (35.6)	0.308
One or more diseased vessels, n (%)	231 (39.1)	827 (40.7)	0.551
Left Main Stem Disease, n (%)	18 (3.05)	85 (4.18)	0.282
Non-elective Procedure, n (%)	120 (20.3)	245 (12.1)	<0.001
Access Site			
Transfemoral, n (%)	454 (76.8)	1673 (82.3)	0.004
Transapical, n (%)	80 (13.5)	169 (8.31)	<0.001
Subclavian, n (%)	23 (3.89)	62 (3.05)	0.374
Other, n (%)	33 (5.58)	127 (6.25)	0.623

** Defined as creatinine >200µmol/l or dialysis for renal failure. LVEF: Left Ventricular Ejection Fraction, MI: Myocardial Infarction, NYHA: New York Heart Association, PCI: Percutaneous coronary intervention.*

For peer review only

Supplementary Table 5. Multivariable adjusted short-term outcomes for the sensitivity analysis of the composite frailty indicator. Bold items indicate significant results.

**Composite Frailty**	**Moderately frail vs. non-frail multivariable OR (95% CI) ***	**Severely frail vs. non-frail multivariable OR (95% CI) ***
30-day mortality	1.19 (0.74, 1.92)	**2.99 (1.71, 5.23)**
Early Safety	1.18 (0.91, 1.52)	**1.88 (1.32, 2.68)**

**: Severely Frail (N=297) corresponds to a patient defined as frail across all three of CSHA, KATZ and poor mobility; Moderately Frail (N=1169) corresponds to a patient that is defined frail by at least one (but not all) of CSHA, KATZ and poor mobility; Non-frail (N=1158) means a patient was defined as not frail by all of CSHA, KATZ and poor mobility.*

CI: Confidence Interval, MVC: Major Vascular Complication, OR: Odds Ratio, PPM: Permanent Pacemaker Implantation

For peer review only

Supplementary References

1 Janssen KJM, Moons KGM, Kalkman CJ, *et al.* Updating methods improved the performance of a clinical prediction model in new patients. *J Clin Epidemiol* 2008;**61**:76–86. doi:10.1016/j.jclinepi.2007.04.018

2 Steyerberg EW, Borsboom GJJM, van Houwelingen HC, *et al.* Validation and updating of predictive logistic regression models: a study on sample size and shrinkage. *Stat Med* 2004;**23**:2567–86. doi:10.1002/sim.1844

3 Moons KGM, Kengne AP, Grobbee DE, *et al.* Risk prediction models: II. External validation, model updating, and impact assessment. *Heart* 2012;**98**:691–8. doi:10.1136/heartjnl-2011-301247

4 Su T-L, Jaki T, Hickey GL, *et al.* A review of statistical updating methods for clinical prediction models. *Stat Methods Med Res* Published Online First: 26 July 2016. doi:10.1177/0962280215626466

For peer review only

STROBE 2007 (v4) checklist of items to be included in reports of observational studies in epidemiology*
Checklist for cohort, case-control, and cross-sectional studies (combined)

Section/Topic	Item #	Recommendation	Reported on page #
**Title and abstract**	1	(*a*) Indicate the study's design with a commonly used term in the title or the abstract	Title page
		(*b*) Provide in the abstract an informative and balanced summary of what was done and what was found	2
**Introduction**			
Background/rationale	2	Explain the scientific background and rationale for the investigation being reported	5
Objectives	3	State specific objectives, including any pre-specified hypotheses	5
**Methods**			
Study design	4	Present key elements of study design early in the paper	6
Setting	5	Describe the setting, locations, and relevant dates, including periods of recruitment, exposure, follow-up, and data collection	6, 7 and 8
Participants	6	(*a*) *Cohort study*—Give the eligibility criteria, and the sources and methods of selection of participants. Describe methods of follow-up<br>*Case-control study*—Give the eligibility criteria, and the sources and methods of case ascertainment and control selection. Give the rationale for the choice of cases and controls<br>*Cross-sectional study*—Give the eligibility criteria, and the sources and methods of selection of participants	6
		(*b*) *Cohort study*—For matched studies, give matching criteria and number of exposed and unexposed<br>*Case-control study*—For matched studies, give matching criteria and the number of controls per case	N/A
Variables	7	Clearly define all outcomes, exposures, predictors, potential confounders, and effect modifiers. Give diagnostic criteria, if applicable	6 and 7
Data sources/ measurement	8*	For each variable of interest, give sources of data and details of methods of assessment (measurement). Describe comparability of assessment methods if there is more than one group	*6*
Bias	9	Describe any efforts to address potential sources of bias	7 and 8
Study size	10	Explain how the study size was arrived at	N/A – registry data
Quantitative variables	11	Explain how quantitative variables were handled in the analyses. If applicable, describe which groupings were chosen and why	7 and 8
Statistical methods	12	(*a*) Describe all statistical methods, including those used to control for confounding	7 and 8
		(*b*) Describe any methods used to examine subgroups and interactions	7 and 8
		(*c*) Explain how missing data were addressed	7
		(*d*) *Cohort study*—If applicable, explain how loss to follow-up was addressed<br>*Case-control study*—If applicable, explain how matching of cases and controls was addressed	N/A

For peer review only

		Cross-sectional study—If applicable, describe analytical methods taking account of sampling strategy	
		(*e*) Describe any sensitivity analyses	N/A
**Results**			
Participants	13*	(a) Report numbers of individuals at each stage of study—eg numbers potentially eligible, examined for eligibility, confirmed eligible, included in the study, completing follow-up, and analysed	9
		(b) Give reasons for non-participation at each stage	9
		(c) Consider use of a flow diagram	N/A
Descriptive data	14*	(a) Give characteristics of study participants (eg demographic, clinical, social) and information on exposures and potential confounders	Table 2
		(b) Indicate number of participants with missing data for each variable of interest	9
		(c) *Cohort study*—Summarise follow-up time (eg, average and total amount)	N/A
Outcome data	15*	*Cohort study*—Report numbers of outcome events or summary measures over time	Table 5 and throughout results section
		Case-control study—Report numbers in each exposure category, or summary measures of exposure	N/A
		Cross-sectional study—Report numbers of outcome events or summary measures	N/a
Main results	16	(*a*) Give unadjusted estimates and, if applicable, confounder-adjusted estimates and their precision (eg, 95% confidence interval). Make clear which confounders were adjusted for and why they were included	Table 5, Table 6 and page 11
		(*b*) Report category boundaries when continuous variables were categorized	N/A
		(*c*) If relevant, consider translating estimates of relative risk into absolute risk for a meaningful time period	N/A
Other analyses	17	Report other analyses done—eg analyses of subgroups and interactions, and sensitivity analyses	Table 7 and throughout results section
**Discussion**			
Key results	18	Summarise key results with reference to study objectives	13
Limitations	19	Discuss limitations of the study, taking into account sources of potential bias or imprecision. Discuss both direction and magnitude of any potential bias	16 and 17
Interpretation	20	Give a cautious overall interpretation of results considering objectives, limitations, multiplicity of analyses, results from similar studies, and other relevant evidence	14, 15
Generalisability	21	Discuss the generalisability (external validity) of the study results	15
**Other information**			
Funding	22	Give the source of funding and the role of the funders for the present study and, if applicable, for the original study on which the present article is based	17

For peer review only

*Give information separately for cases and controls in case-control studies and, if applicable, for exposed and unexposed groups in cohort and cross-sectional studies.
Note: An Explanation and Elaboration article discusses each checklist item and gives methodological background and published examples of transparent reporting. The STROBE checklist is best used in conjunction with this article (freely available on the Web sites of PLoS Medicine at http://www.plosmedicine.org/, Annals of Internal Medicine at http://www.annals.org/, and Epidemiology at http://www.epidem.com/). Information on the STROBE Initiative is available at www.strobe-statement.org.

For peer review only
